# Label-free Aβ plaque detection in Alzheimer's disease brain tissue using infrared microscopy and neural networks

**DOI:** 10.1016/j.heliyon.2025.e42111

**Published:** 2025-01-18

**Authors:** Dajana Müller, Dominik Röhr, Baayla D.C. Boon, Maximilian Wulf, Thomas Arto, Jeroen J.M. Hoozemans, Katrin Marcus, Annemieke J.M. Rozemuller, Frederik Großerueschkamp, Axel Mosig, Klaus Gerwert

**Affiliations:** a Ruhr University Bochum, Center for Protein Diagnostics (PRODI), Bioinformatics Division, Germany; b Ruhr University Bochum, Faculty of Biology and Biotechnology, Department of Bioinformatics, Germany; c Ruhr University Bochum, Center for Protein Diagnostics (PRODI), Biospectroscopy Division, Germany; d Ruhr University Bochum, Faculty of Biology and Biotechnology, Department of Biophysics, Germany; e Amsterdam UMC, Amsterdam Neuroscience, Department of Pathology, the Netherlands; f Mayo Clinic, Department of Neuroscience, Jacksonville, FL, USA; g Ruhr University Bochum, Center for Protein Diagnostics (PRODI), Medical Proteome Analysis, Germany; h Ruhr University Bochum, Faculty of Medicine, Medizinisches Proteom-Center, Germany

**Keywords:** Alzheimer's disease, Amyloid-beta plaque, Human, Microspectroscopy, Infrared, Imaging, Deep learning, Neural networks, Proteomics

## Abstract

We present a novel method for the label-free detection of amyloid-beta (Aβ) plaques, the key hallmark of Alzheimer's disease, in human brain tissue sections. Conventionally, immunohistochemistry (IHC) is employed for the characterization of Aβ plaques, hindering subsequent analysis. Here, a semi-supervised convolutional neural network (CNN) is trained to detect Aβ plaques in quantum cascade laser infrared (QCL-IR) microscopy images. Laser microdissection (LMD) is then used to precisely extract plaques from snap-frozen, unstained tissue sections. Mass spectrometry-based proteomics reveals a loss of soluble proteins in IHC stained samples. Our method prevents this loss and provides a novel tool that expands the scope of molecular analysis methods to chemically native plaques. Insight into soluble plaque components will complement our understanding of plaques and their role in Alzheimer's disease.

## 1 Introduction

Alzheimer's disease (AD) is a devastating and prevalent neurodegenerative disease that affects more than 35 million people worldwide, which is predicted to rise up to 115 million cases by 2050 [1]. The main neuropathological hallmark of AD is the accumulation of Aβ plaques in the brain. The well-established, but hotly debated, amyloid cascade hypothesis posits that an imbalance between Aβ production and clearance leads to the extracellular accumulation of Aβ [[2], [3], [4]]. Aβ monomers misfold to form β-sheet-rich oligomers and fibrils that aggregate as plaques [[5], [6], [7]]. The aggregated Aβ is proposed to cause synaptic stress, tau hyperphosphorylation, and neuronal injury that eventually results in neuronal death [2]. As AD progresses, there is widespread neurodegeneration throughout the brain, which ultimately leads to dementia.

Traditionally, Aβ plaques are detected in brain tissue sections using stains like Congo red, Thioflavin S or anti-Aβ IHC, e.g. to study the plaque morphology or to perform neuropathological diagnosis. Such staining methods involve the use of various solvents, detergents and other reagents that alter the chemical composition of the sample. This may impose a yet uncharacterized bias onto downstream analysis. Here, we present a novel label-free plaque detection method and use it to investigate the influence of IHC onto the tissue proteome.

In recent years, spectral imaging techniques, such as infrared (IR) imaging, have been explored to simultaneously investigate the morphology and chemical composition of tissues. IR imaging obtains high-resolution hyperspectral images of tissue sections without the need to process the tissue, preserving the tissue in its chemically native state. This facilitates a chemically unbiased investigation and makes unaltered samples available for further downstream analysis. IR imaging has been applied by several groups for the classification of cancerous tissues [[8], [9], [10], [11], [12]]. For example, the identification, subtyping, grading, and molecular alterations of colon and lung cancer has been reported by our group [[13], [14], [15], [16], [17], [18], [19], [20]]. The introduction of quantum cascade lasers (QCLs) as light sources in IR imaging has greatly reduced measurement, enabling rapid analysis of whole tissue sections [[19], [20], [21], [22], [23], [24]]. The combination with LMD allows for the precise extraction and molecular analysis of pathologic tissue regions [16,19,25].

A label-free detection and subsequent extraction of Aβ plaques in AD is desirable because it allows the unimpeded investigation of sensitive tissue components, such as soluble proteins, lipids, and unstable Aβ oligomers. Aβ plaques in AD have been investigated with IR imaging by several groups, yielding insights into the protein secondary structure and lipid composition of plaques [[26], [27], [28], [29], [30]]. Our group previously demonstrated that mean IR spectra of plaques differ significantly from their surrounding tissue [7]. However, individual pixel spectra alone are insufficient for reliably identifying Aβ-positive regions, as we have demonstrated ourselves in a preliminary study, detailed in the supplementary material (Supplementary Fig. S1). This is presumably due to the significant chemical heterogeneity within plaques [31,32]. This variability underscores the need for a detailed chemical characterization of plaques. Given these challenges, IR imaging is particularly promising as it may allow for the label-free detection of plaques based on their chemical composition.

Machine learning approaches, particularly neural networks (NNs), have emerged as powerful tools in the field of digital pathology [33], enabling automated classification and segmentation tasks. NNs, inspired by the interconnectedness of neurons in the human brain, have the ability to learn complex patterns and relationships within large-scale datasets which makes them particularly well-suited for the analysis of medical images. The combination of IR microscopic images with deep CNNs has significantly enhanced the ability to identify and to precisely localize pathological tissue regions [20,34]. In the realm of AD pathology, the integration of NNs with spectral imaging data offers a unique opportunity to leverage the advantages of both techniques, thereby holding great promise for advancing plaque detection and characterization. Here, we train a weakly supervised CNN to detect plaques in QCL-IR images. To the best of our knowledge, our current work comprises the first successful label-free detection of Aβ plaques with an accuracy comparable to that of the established gold standard IHC.

In this paper, we present a novel framework that combines QCL-IR and CNNs for the automated detection of Aβ plaques in post mortem AD brain tissue allowing for unimpeded downstream analysis. The proteome of label-free plaques was compared to that of plaques from IHC stained tissue. As expected, we found a significantly reduced abundance of soluble proteins that were apparently lost during the IHC procedure. This emphasizes that the label-free detection of Aβ plaques harbors a great potential for further unraveling the molecular composition of plaques. This is crucial for understanding the role of Aβ plaques in AD pathogenesis and may enable the discovery of new biomarkers and therapeutic targets in AD.

## 2 Results

### 2.1 QCL-IR and anti-Aβ IHC images serve as training data for the CNN

The CNN is trained with label-free QCL-IR and corresponding anti-Aβ IHC images. The latter guide the selection of suitable regions of interest (ROIs) and are used during the training process. The final CNN only requires label-free QCL-IR images to detect plaques in unstained tissue sections.

**Fig. 1:**
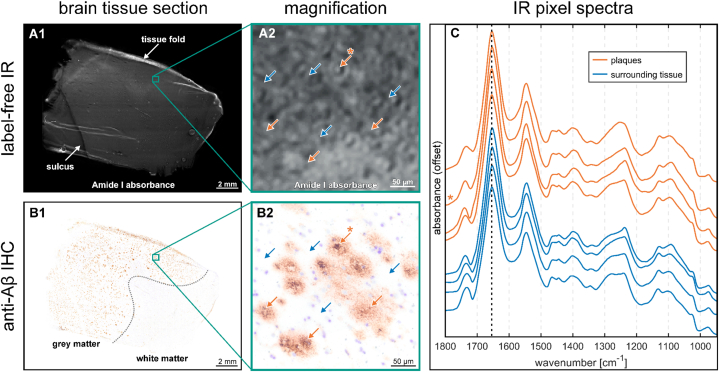
Quantum cascade laser infrared (QCL-IR) and immunohistochemistry (IHC) imaging data. **A1** QCL-IR image of a brain tissue section. Pixel values correspond to the IR absorbance of the Amide I band. **A2** Magnification of the QCL-IR image. Some plaques are marked with orange arrows and a selected classic cored plaque is indicated with an asterisk. Some Aβ-free areas are marked with blue arrows. **B1** Anti-Aβ IHC image of the same section, stained and scanned after QCL-IR experiments. Aβ plaques are visible as brown dots. **B2** Magnification of the IHC image, corresponding to A2. **C** QCL-IR pixel spectra from plaques (orange) and Aβ-free tissue (blue). The corresponding pixels are indicated by arrows in A2 and the spectrum of the selected classic cored plaque is indicated with an asterisk. The spectra are offset by constant values for better visibility.

Fig. 1 provides an overview of the label-free QCL-IR and IHC images. Both are high-resolution, whole-slide microscopy images of brain tissue sections, which are several centimeters in width and height. Fig. 1A1 displays a QCL-IR image of such a section, visualized by the absorbance of the Amide I band of the protein backbone around 1655 cm^−1^. Tissue folds from the sectioning procedure appear as white lines. The dark line on the lower left is a sulcus; a brain fold where two gyri converge. After QCL-IR imaging, the sections are immunostained against Aβ, scanned with brightfield microscopy and precisely aligned with the QCL-IR image [7]. In Fig. 1B1, the plaques are visible as brown dots, mostly within the gray matter region in the left and upper perimeter of the sample. Fig. 1A2 and 1B2 show magnifications of a ROI containing a cluster of plaques. The QCL-IR image in Fig. 1A2 is dominated by the tissue morphology, which only allows the distinction of a few objects. One of them is a classic cored plaque, faintly recognizable as a white dot, surrounded by a ring (orange asterisk). In the IHC image (Fig. 1B2), several plaques are visible, including the aforementioned classic cored plaques that are easily recognizable by their dark brown cores, surrounded by a light brown corona [35,36]. Additionally, compact, and diffuse plaques are visible [35,37]. Aβ-free tissue appears white, sprinkled with violet dots from the cresyl violet counterstain. Fig. 1C depicts QCL-IR pixel spectra from plaques and Aβ-free areas, corresponding to the arrows in Fig. 1B2 and 1C2. The spectral differences between the pixel spectra are subtle, but noticeable variations are observed (i) around the ester band at 1740 cm^−1^, (ii) in the C-H deformations bands between 1480 and 1430 cm^−1^, and (iii) in the region 1320 to 1200 cm^−1^, where multiple IR absorbance bands overlap [38,39]. A high variability in Aβ plaque spectra is observed both across and within cases (Supplementary Fig. S2), with some pixel spectra of plaques closely resembling the mean spectra of the Aβ-free regions. Our deep learning approach aims to utilize the presented spectral differences and spatial image information to detect plaques in QCL-IR images.

### 2.2 An optimized workflow yields large quantities of training data

**Fig. 2:**
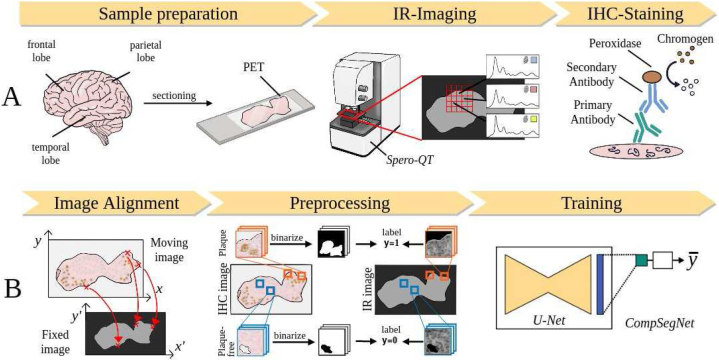
Workflow for training data generation. **A** Experimental phase: Snap-frozen tissue of the frontal lobe, the temporal lobe, and the parietal lobe is cut into 10 μm sections and mounted on Polyethylene terephthalate (PET) slides. QCL-IR Imaging is performed and then the same sample is immunohistochemically stained for Aβ and scanned in a light microscope. **B** Computer-based phase: Precise alignment of IHC images onto the QCL-IR coordinate system. Regions of interest (ROIs) of size 64 × 64 pixels were selected. Binary masks were generated from the corresponding IHC ROIs. The dataset was divided at case level to train a weakly supervised neural network called Comparative Segmentation Network (CompSegNet) on binary image labels.

To create large quantities of training data for the CNN, we developed a comprehensive workflow. It facilitates (i) the production of suitable tissue samples, (ii) the measurement of whole-slide QCL-IR images, (iii) IHC staining against Aβ followed by brightfield microscopy, (iv) the precise alignment of both images, (v) the selection of ROIs, and (vi) their preprocessing. Fig. 2 gives an overview of the workflow, whereas a detailed description of each step can be found in the method section. Fig. 2A depicts the experimental phase of the procedure. First, snap-frozen tissue blocks of the frontal lobe, temporal lobe, and parietal lobe are sectioned at 10 μm and thaw-mounted on Polyethylene terephthalate (PET) slides. The sections are imaged with the QCL-IR microscope Spero-QT (Daylight Solutions) and subsequently stained against Aβ using indirect chromogenic IHC. The stained sample is scanned with a brightfield microscope (BX61, Olympus). The computational portion of the workflow is depicted in Fig. 2B. First, a custom software is used to determine an affine transformation that precisely maps the IHC image onto the QCL-IR image coordinates [7]. Then, IHC ROIs are selected and binarized, with Aβ-positive pixels being 1 and Aβ-free pixels being 0. Each ROI is labeled accordingly as Aβ-containing (y = 1) or Aβ-free (y = 0). A CNN called Comparative Segmentation Network (CompSegNet) [34] is trained on these binary ROI labels to identify Aβ plaques in QCR-IR images.

### 2.3 The neural network architecture for Aβ plaque detection is a semi-supervised framework

**Fig. 3:**
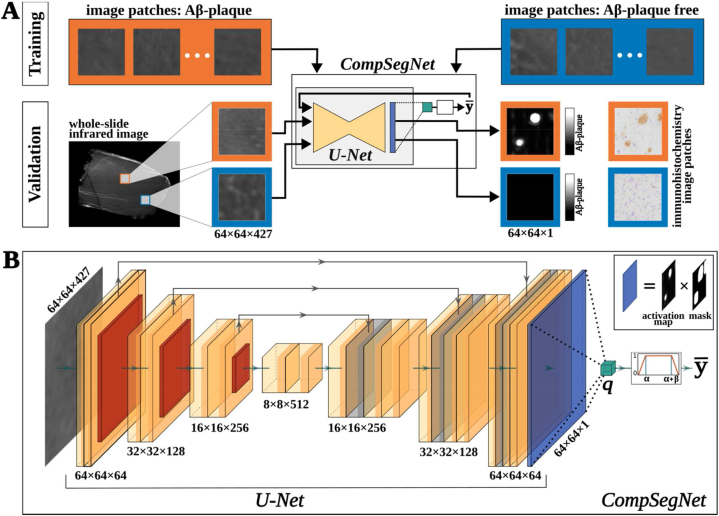
Overview of the Comparative Segmentation Network (CompSegNet) and the training process. **A** The network is trained on pairs of infrared (IR) regions of interest (ROIs) and binary image labels. ROIs can be amyloid-beta (Aβ)-free (*y = 0*) or contain plaques (*y = 1*). **B** CompSegNet consists of a depth-reduced U-Net with an adapted input layer of size 64 × 64 × 427, yielding a 64 × 64 × 1 activation map which is connected to a pooling neuron *q.* The transfer function will maximize activation in Aβ-positive ROIs and minimize activation in Aβ-free areas.

The CompSegNet is a semi-supervised approach which is trained on binary labels to overcome the need for a pixel precise annotation. It infers a segmentation of Aβ plaques during training while using problem-specific background masks. Fig. 3A illustrates the training process of the CNN. The input comprises a dataset of ROIs of size 64 × 64 × 427, corresponding to 269 × 269 μm of tissue, and consists of Aβ plaque (orange) and Aβ-free (blue) images. The output is an activation map, a grayscale image that indicates the predicted potentiality that a plaque is present. The training process aims to infer an activation map by localizing Aβ structures in an Aβ positive sample. For a positive ROI (y = 1), the output map should approximate a value of 1 for positions where Aβ is present and 0 for Aβ-free pixels. For a ROI with label y = 0, activation should approach 0 across all pixels. During the training process, the network is validated using a separate dataset which is distinct from the training data to evaluate the model's performance and its generalization ability. The CompSegNet consists of a depth-reduced U-Net [40] architecture, in which an QCL-IR image ROI is processed through a series of down- and up-convolution layers to produce an activation map of the same spatial dimensions as the input image. Each activation map is then weighted by their binary mask, so that Aβ-free background positions are set to 0 and then averaged in the connected pooling neuron *q* that is regulated by a characteristic transfer function (Fig. 3B). The purpose of the transfer function is to guide the learning process towards identifying a certain amount of Aβ in positive ROIs (α < Aβ < α + β) and minimizing the activation in Aβ-free images while using two thresholds. By utilizing the pooling neuron, the segmenting network effectively becomes a binary classifier, where the classification accuracy depends on the segmentation output from the activation layer. The final loss is backpropagated through the network and enables the learning process. The loss function consists of two terms: a background loss which penalizes activated pixels which are masked out or belong to the Aβ-free group and a class-loss which weights the two classes based on the amount of the relative input patches in the dataset.

### 2.4 The NN is able to generalize and shows high specificity on hold-out data

**Fig. 4:**
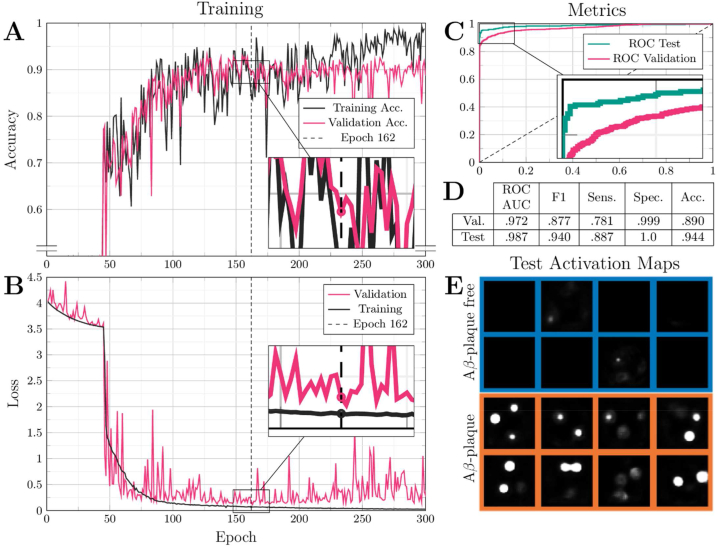
Overview of training and evaluating CompSegNet trained on infrared ROIs. **A** Loss curves are shown in gray for training data and in magenta for validation data over 300 epochs. **B** Accuracy curves during training. **C** Comparison of receiver operating characteristic (ROC) curves for validation (magenta) and testing data (green). **D** Statistical metrics for the selected model from the 162nd epoch. **E** Random selection of activation maps of Aβ-free (blue) and Aβ-positive (orange) testing data.

CompSegNet was trained on hyperspectral data for 300 epochs. Fig. 4A illustrates the loss curves for the training and validation dataset with an initial plateau phase which is characteristic for the CompSegNet. Afterwards, the training loss decreased steadily and converged around epoch 150 with only marginal fluctuations (Fig. 4A Zoom-In). Epoch 162 (dotted line) was selected based on the highest specificity, taking into account accuracy (Fig. 4B), sensitivity, specificity, F1 score and area under receiver operating characteristic curve (AUC-ROC) (Fig. 4C) on the validation dataset (Fig. 4D). The model's (epoch 162) ability to generalize to unseen data was assessed by validating its performance on an hold-out test set (∑n = {3 AD patients, 1 non-AD patient}, Table 1). Fig. 4C and D shows similar results for validation and test set, with an AUC-ROC value of 0.987 and a F1-score of 0.94 on the test data, indicating a generalized and stable model. A random selection of activation maps of test ROIs is shown in Fig. 4E. Blue background imply Aβ-free samples and orange background Aβ-positive, respectively. In Aβ-free ROIs, only a minimal amount of activation was observed while in plaque-containing ROIs, circular activation structures that are indicative for plaques can be found.

**Table 1:** Details on group demographics, neuropathological staging and dataset compositions. Neuropathological scoring for Aβ deposits (A) and neurofibrillary tangles (B) [65]. Abbreviations: AD Alzheimer's disease, HC healthy control, m male, f female, ROI region of interest.

	Training (n = 10)		Validation (n = 5)		Testing (n = 4)		∑ (n = 19)		
	AD	HC	AD	HC	AD	HC	AD	HC	All
**Cases**	9	1	4	1	3	1	16	3	19
**sex (**m/f)	6/3	0/1	1/3	1/0	2/1	0/1	9/7	1/2	10/9
**age at death** (mean ± SD)	71 ± 15	78	73 ± 10	83	78 ± 9	102	73 ± 13	88 ± 10	75 ± 14
**A (amyloid)** n per stage 0/1/2/3	0/0/0/9	0/1/0/0	0/0/0/4	0/1/0/0	0/0/0/3	0/1/0/0	0/0/0/16	0/3/0/0	0/3/0/16
**B (tau)** n per stage 0/1/2/3	0/0/2/7	0/1/0/0	0/0/0/4	0/1/0/0	0/0/0/3	0/1/0/0	0/0/2/14	0/3/0/0	0/3/2/14
**Samples**	44	2	13	2	3	1	60	5	65
- temporal lobe	11	–	6	–	2	1	19	1	20
- frontal lobe	18	–	–	–	1	–	19	0	19
- parietal lobe	15	2	7	2	–	–	22	4	26
**ROIs** Aβ-positive	4702	–	1241	–	372	–	6315	–	6315
Aβ-free	3410	1292	886	355	181	191	4477	1838	6315

### 2.5 Strong alignment between the NN's segmentation and the IHC staining

**Fig. 5:**
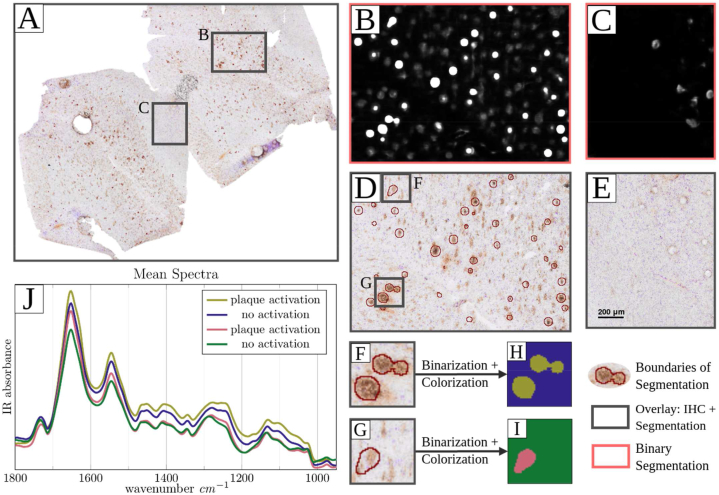
Results for whole-slide segmentation of model 162 on an hold-out test sample. **A** Overlay of the immunohistochemistry image and segmentation output with boundary tracing in red. **B/C** Output activation maps of the U-Net of area D and E. **F** and **G** show a second magnification of areas in B with several plaques. **D/E** Magnification of selected regions with (B) and without (C) plaques. **H/I** Colored activation map; activation in olive (H) and rose (I), no activation in blue (H) and green (I). **J** Averaged infrared spectra corresponding to their colorized activation map (H/J).

The segmentation results on whole-slide images (WSI) with Aβ-plaque pathology is presented in Fig. 5. Fig. 5A shows the overlay of a whole-slide segmentation of a test case with boundary tracing (red) and the corresponding IHC image. The plaque segmentation in two adjacent sections is illustrated by comparing the activation maps of plaque-rich gray matter and white matter without plaques. A magnification of a selected region in which plaques are present is visualized in 5D and the corresponding area of the activation map in 5B. Regions with brighter white pixels indicate plaques, while regions with darker pixels suggest a lower likelihood of plaque presence. The activation map reveals that denser Aβ plaques, which appear darker in the IHC, were more consistently recognized by the network compared to the lighter structures (Supplementary Fig. S3). Additionally, the activation map exhibited predominantly round shapes that closely resembled the shape of the Aβ plaques in the IHC. In addition, spectral differences were examined between the Aβ-positive pixels detected by the network and the background with little to no activation (Fig. 5F and G), to gain further insights into the network's performance in identifying Aβ plaques. Therefore, the activation map of two different areas were colorized depending on their activation level (H,I) and the corresponding spectra of each colored area were averaged and plotted in 5J. Subtle spectral differences between the activation of the dense core plaque (H, olive) and its surroundings (H, blue) can be observed throughout the whole spectral range, whereas spectral differences between the activation of a diffuse plaque (I, rose) and the background (I, green) can only be found in the Amid I (1655 cm^−1^) and II bands (1545 cm^−1^). To facilitate comparison, a magnified view of an Aβ-free area is shown in 5E, alongside the corresponding activation map in 5C. Further evaluation of our model's performance on the validation and test datasets is available in the Supplementary Figs. S3 and S4. These Figures contain activation maps segmented by case, facilitating an in-depth analysis of the model's behavior across different subjects. Notably, in Fig. 5D and Supplementary Fig. S4, the network more frequently detects plaques with more intense IHC staining, compared to more diffuse structures which appear lighter and amorphous in the IHC ROIs.

### 2.6 The label-free plaque detection is robust and enables the precise extraction of plaques

**Fig. 6:**
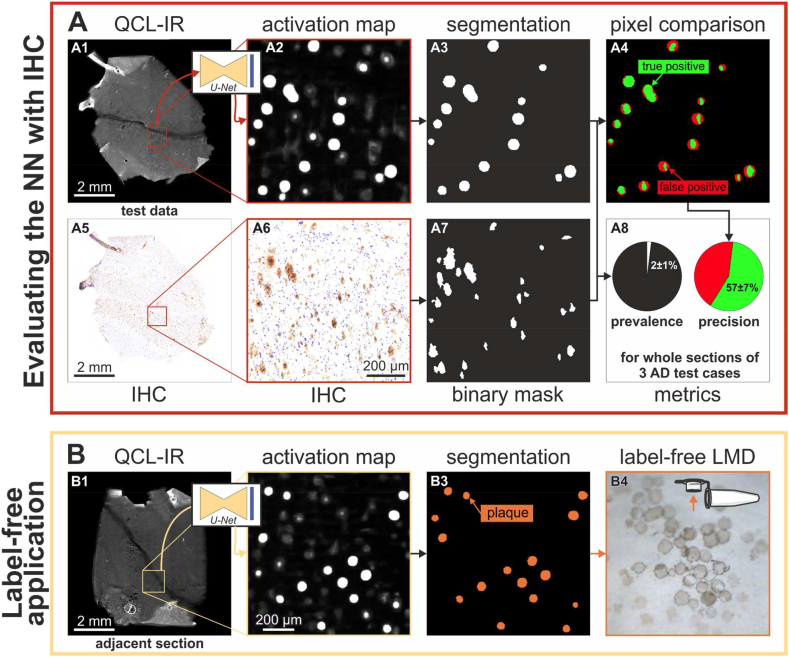
Evaluation of the segmentation performance and subsequent label-free plaque extraction. **A** The NN processes QCL-IR images of test case samples (A1) into activation maps (A2) that are binarized to segment predicted plaques (A3). IHC images of the same section (A5 and A6) are binarized for comparison (A7). A4 Pixel-by-pixel comparison between the NN prediction (red) and IHC (green) facilitates the calculation of the segmentation precision (A8). **B** An activation map is generated from a QCL-IR image of an adjacent section (B1 and B2). The activation map is segmented to determine predicted plaques shapes (B3, orange). The shapes (B4) are extracted using laser microdissection (LMD) and collected in buffer drops in tube lids.

To quantify the segmentation performance of the NN, whole-slide activation maps were compared to their corresponding IHC images, as illustrated in Fig. 6A. The NN processes whole-slide QCL-IR images (6A1) into activation maps (6A2) that are binarized (6A3) and compared to the corresponding IHC images (6A4 to 6A7). The precision of segmentation is determined by pixel-by-pixel comparison of the resulting binary images. This procedure was performed on samples from all AD cases (n = 3) in the test dataset. The calculated precision of 57 ± 7 % depicts the proportion of actual plaque area (green) within the predicted plaque area (red), as depicted in Fig. 6A4. Fig. 6A8 displays the average plaque prevalence (plaque area per sample area) in the test samples at 2 ± 1 %. In contrast, the NN precision (true positive area per positive area) is 57 ± 7 %. The ratio between NN precision and prevalence is defined here as the *purification factor PF*, which indicates the factor by which plaques can be purified, relative to a homogenized tissue section. The average purification factor is 32 ± 17.

The plaque detection in QCL-IR images is used to extract plaques from unstained adjacent tissue sections, as depicted in Fig. 6B. First, a scanned QCL-IR image of a section (6B1) is processed into a whole-slide activation map (6B2), which is used to segment predicted plaques (6B3). The sample and the shape coordinates are transferred to an LMD microscope. There, the plaques are extracted and collected in buffer drops in tube lids (6B4). This way, several hundred label-free plaques, equivalent to 10^6^ μm^2^ tissue area, were extracted from each section.

### 2.7 IHC alters the proteome of Aβ plaques

**Fig. 7:**
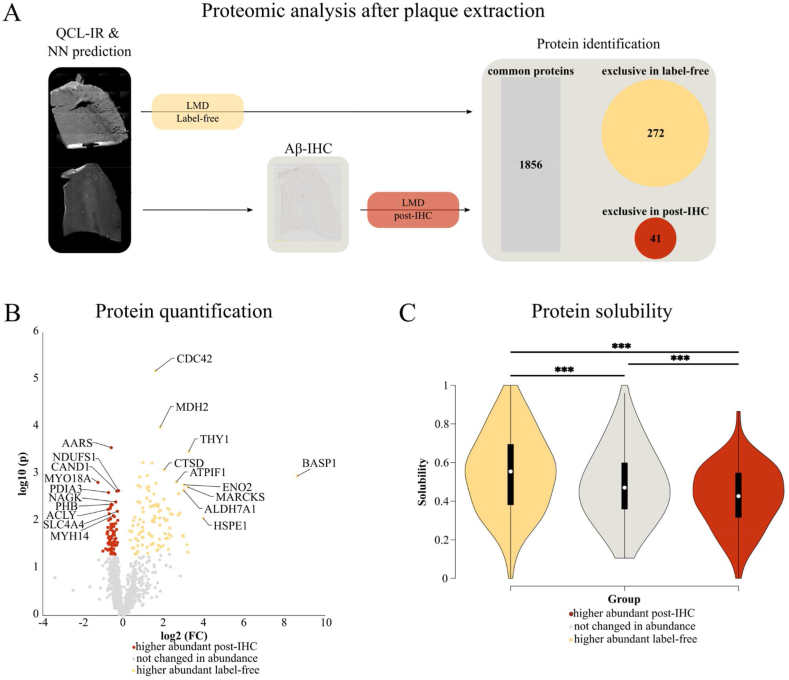
Comparing the proteome of label-free and post-IHC Aβ plaques. **A** Plaques were extracted using laser microdissection (LMD) according to quantum cascade laser infrared (QCL-IR) imaging and neural network plaque detection. Tissue samples from AD test cases (n = 3) either remained label-free (N = 3) or were extracted after immunohistochemical staining (post-IHC; N = 3). Our complete dataset comprises 2168 proteins, of which 1856 proteins (gray) were identified in both groups. Label-free plaques yielded higher numbers of exclusively identified proteins (272, yellow) than post-IHC plaques (41, red). **B** Of 906 proteins matching criteria for quantitative comparison, 102 proteins were significantly higher abundant in label-free samples (yellow), while 73 proteins were significantly higher abundant in post-IHC samples (red). **C** Protein solubilities scores [43] were significantly different between the three abundance groups (∗∗∗: p-value <0.001).

The proteome of Aβ plaques is essential for understanding the role of plaques in AD pathogenesis and can be used to screen for potential biomarkers [41]. We compare the proteome of plaques from unstained tissue sections (label-free) with plaques from anti-Aβ IHC stained adjacent sections (post-IHC), as shown in Fig. 7A. In both cases plaques were extracted according to QCL-IR-based CNN predictions, ensuring highly comparable samples. This was done for sequential tissue sections of the three AD cases in the hold-out test group, described in Section 2.4. The extracted plaques, both label-free and post-IHC, were investigated with bottom-up proteomics [42]. 1856 proteins were present in both groups with comparable abundances (Supplementary Fig. S5), including well-known plaque proteins as Aβ, tau and proteins of the complement system. 272 proteins were found exclusively in label-free plaques, including various lysosomal proteins, whereas post-IHC plaques contained only 41 exclusive proteins (Fig. 7A, Supplementary Fig. S6A, Supplementary Table ST1). Further, we found that fold changes of protein abundance displayed a wider dynamic range in label-free plaques than in post-IHC, with the highest fold change being 393-fold for *brain acid soluble protein 1* (BASP1), as seen in Fig. 7B. In contrast, the fold changes in post-IHC plaques only reach up to 2.4, emphasizing the benefit of label-free extraction for proteomic analysis. We hypothesized that especially highly soluble proteins would be lost during IHC. Indeed, we found that the proteins with higher abundances in label-free plaques are significantly more soluble than those which are higher abundant in post-IHC plaques, as shown in Fig. 7C.

## 3 Discussion

Label-free imaging techniques, such as IR and Raman imaging, have been used by various research groups to study the composition of plaques [[27], [28], [29], [30],[44], [45], [46], [47], [48]]. Further, the autofluorescent properties of plaques were studied by several groups [[49], [50], [51], [52]]. Lochocki and Ettema et al. found that autofluorescence, combined with the Raman signature of β-sheets and carotenoids, are markers of cored plaques [[53], [54], [55]]. However, they state that these markers only identified a subset of plaques with very high levels of amyloid, limiting the use of these markers for label-free plaque detection.

In addition to these findings, research has shown significant variability in the spectral characteristics of plaques. Confer et al. demonstrated that even morphologically similar plaques exhibit pronounced spectral variability, particularly in their secondary structure and phospholipid content [31]. Similarly, Holcombe et al. (2023) highlighted plaque-to-plaque variability in the β-sheet structures within cored plaques, finding both parallel and antiparallel β-sheets, with some plaques lacking the distinctive spectral features from their surroundings [32]. This underscores that using the entire spectrum is crucial for accurately distinguishing Aβ plaques from adjacent tissue, as focusing solely on protein bands may not capture the full range of chemical differences necessary for effective identification. At the same time, the spectral differences have a spatial-morphological component, as plaques are local accumulations that exhibit the relative spectral differences to the surrounding tissue. This underlines the importance of utilizing convolutional neural networks, which can learn such combinations of spectral and spatial alterations. Our work involves spectra from overall sixteen AD patients, and thus provides a more general picture of the spectral and spatial alterations that characterize Aβ plaques than previous contributions which involved much smaller patient numbers. To capture the heterogeneity across patients, our work involves a convolutional neural network, which can capture the spatial accumulations of spectral changes.

In contrast, the approach presented in this study represents the first successful label-free detection of plaques by leveraging the potential of deep learning, providing a promising alternative to label-based methods like IHC. The performance of our label-free approach was evaluated by the comparison to anti-Aβ IHC staining on the same tissue section which is currently considered the gold standard for plaque detection in neuropathology. Our classification results per image patch demonstrated high accuracy and sensitivity on the validation and hold-out test samples (Fig. 4) The segmentation results revealed the effective identification of plaques by our method and showcased its ability to successfully detect Aβ-plaques at pixel-level precision of 57 %. Thus, closely approximating the IHC gold standard and allowing to purify plaques 32-fold using NN-guided LMD. These evaluation metrics collectively demonstrate the effectiveness and reliability of our label-free plaque detection approach.

The label-free approach holds great promises for plaque detection in AD research. By overcoming the need for immunohistochemical markers, this method provides a valuable tool for analyzing plaques and opens avenues for investigating the molecular composition of unaltered plaques. However, it is crucial to acknowledge the limitations and biases associated with the neural network approach. Firstly, the spatial pixel resolution of IR imaging is limited to above 5 μm within the relevant spectral region. This resolution may not be sufficient to resolve small plaques, resulting in their absence in the plaque extracts generated by the method. Secondly, the NN demonstrates a higher reliability in detecting dense plaques compared to diffuse plaques. Consequently, the composition of the plaque extracts may exhibit disproportionate amounts of classic cored and compact plaques in relation to diffuse plaques. Thirdly, the NN does not fully replicate the results of anti-Aβ IHC at pixel level, which is currently considered the gold standard for plaque detection in neuropathology. This impedes a direct comparison between the results obtained from label-free extraction and label-based extraction techniques.

Another important aspect that has to be considered when analyzing the segmentation results from our NN is the presence of non-Aβ protein aggregates and the potential for false-positive. Following the spectral acquisition, samples were stained against Aβ, and binary masks were created based on the IHC results. The loss function of CompSegNets utilizes these masks and guides the learning process to implicitly identify differences between Aβ-free and Aβ plaque areas through their spectral and morphological characteristics, although the precise mechanisms by which these differentiations occur are not yet fully understood.

The aim of this study is to generate an assay for label-free plaque identification and subsequent chemically unimpeded molecular analysis within a specific set of samples. The model's scientific relevance lies in generating hypotheses about plaque formation mechanisms and an unbiased understanding of plaque characteristics that find support in downstream studies. Therefore, the segmentation performance of the NN on the used cohort (N = 19 cases) takes precedence over generalizing the model to other cohorts. In this context, the need for the model to perform reliably across varying trials and in an everyday medical care setting is less critical, as our focus lies on the possibilities of this workflow. While the performance of our NN on samples from different origins or in other laboratories remains uncertain, experiences from multi-centric studies in histopathology clearly suggest that the models described here would require a certain amount of retraining to adjust to data from a different cohort or a different laboratory [56].

Explainability is a crucial factor to consider in our study. While we successfully utilized deep learning methods for label-free plaque detection, it is important to acknowledge that chemometrics are often the preferred method as NNs are perceived as “black box” approaches, making it challenging to discern the knowledge acquired during training. To address the demand for interpretability in our NN, approaches such as layer-wise relevance propagation [57,58] Grad-CAM [59] or saliency maps [60] are applicable methods in identifying key input features and important wavenumbers that contribute to the decision-making process of the network. These methods can significantly enhance our understanding of various factors and bridge the gap towards a more explainable approach in future studies. However, the chemical signal in infrared spectra obtained from tissue samples is entangled with complex physical effects on the spectrum, and it remains a major research challenge for the field to disentangle chemical information from complex tissue spectra.

The proteomic data presented in our study provide first promising insights into the benefits that label-free plaque extraction provides for proteomic studies. Although only a small cohort was used for proteomic experiments (n = 3), our results show that both protein identification and quantification can be improved by avoiding IHC procedure. These observations need to be further verified with a larger cohort but should nevertheless be considered in the context of the combination of IHC and proteomic investigations. In addition, it might be possible to even further improve the identification and quantification of proteins from plaques by changing the mass spectrometric measurement mode from data-dependent to data-independent acquisition, since this acquisition mode has recently shown to be advantageous regarding protein identification and quantification [61,62].

Although further improvements are still possible, our relatively small dataset showed a high overlap with a previous study focusing on plaque proteomics [63], because our dataset includes 186 of the 279 proteins reported as proteins commonly found in Aβ plaques (Supplementary Fig. S6B). However, it should be noted that Drummond et al. used FFPE tissue with IHC, which is a more invasive method than our IHC staining on fresh frozen tissue, potentially resulting in variations in protein yield due to the differing tissue preparation methods. Additionally, while the availability of fresh frozen tissue is limited, FFPE tissue is far more challenging for sample preparation and data analysis for proteomic studies due to protein cross-linking and altered antigenicity,

Future research endeavors should (i) prioritize the refinement of the technique employed in this study to enhance its spatial resolution, (ii) broaden the sample size for both training and testing purpose and (iii) evaluate its performance across diverse sample populations and different laboratory settings to contribute to its generalizability. Furthermore, exploring the utility of the technique in animal models of AD will offer valuable insights into its effectiveness and potential limitations. A meaningful application of our technique is the investigation of Aβ oligomers in the AD brain, which presents significant challenges, as these oligomers are considered transient and unstable polymorphs that are highly susceptible to their surrounding conditions [64].

In conclusion, the successful implementation of label-free plaque detection using a NN represents a significant breakthrough in the research field of Aβ plaques. To the best of the authors' knowledge, this is the first time such a method has been successfully employed. The label-free detection and extraction of plaques from tissue thin sections using QCL-IR imaging, a NN, and LMD provide a fundamentally novel tool for plaque analysis that we demonstrated to provide a significant benefit. This approach enables the extraction of plaques in their chemically native state without the need of IHC staining potentially altering the plaque proteome, thus allowing for a wide range of analytical methods to be applied to these previously inaccessible samples, opening new possibilities for studying the nature of plaques, particularly their transient constituents. This brings us closer to the in vivo state of plaques, offering a more reliable and unbiased understanding of plaque characteristics that can contribute to advancing our knowledge of this devastating condition. The insights gained from these studies may aid in the development of more effective diagnostic and therapeutic strategies for AD.

## 4 Materials and methods

**Case selection.** AD cases (n = 16) were selected when clinical and neuropathological information fulfilled the criteria of the National Institute on Aging-Alzheimer's Association (NIA-AA) for AD [65]. We selected AD cases with the intention to cover a wide variety of Aβ deposits. This is a crucial prerequisite for a generalizing classifier, which recognizes the universal features of Aβ plaques. Further, we selected both female (n = 9) and male cases (n = 7) with vastly different ages at death, ranging from 37 to 92 years. We selected tissue from three regions of the neocortex, the frontal lobe, the temporal lobe, and the parietal lobe. Some cases displayed vascular Aβ deposits, referred to as cerebral amyloid angiopathy (CAA), which was not included in our data selection routine. Some AD cases displayed atypical Aβ deposits, such as cotton-wool plaques (n = 2) and coarse-grained plaques (n = 3). These atypical plaques feature a notably different morphology than classic Aβ plaques and are therefore a valuable addition to our dataset [36]. Control cases (n = 3) were selected when no cognitive decline was reported during life and AD pathology was absent or ‘low’ according to the NIA-AA criteria [65]. For details see (Table 1).

**Post-mortem human brain tissue.** Post-mortem brain tissue was selected from the Netherlands Brain Bank (the NBB; Amsterdam, The Netherlands). Donors or their next of kin signed informed consent for the usage of brain tissue and clinical information for research purposes. The Institutional Review Board and Medical Ethical Board from the Vrije University Medical Center approved the procedures of the NBB. Neuropathological diagnosis was performed (by A.J.M.R) and was based on multiple (immuno)histochemical staining of diversified brain regions according to the standard operating procedures of the NBB and BrainNet Europe consortium. Snap-frozen tissue sections (10 μm) were mounted on PET frame slides (Leica) for vibrational imaging and subsequent Aβ-IHC. Tissue sections were stored at −80 °C prior to experiments to reduce sample degradation [27].

**Quantum cascade laser infrared imaging (QCL-IR).** IR imaging is a label-free method for obtaining microscopic spectral images of tissue sections. IR spectra are a molecular fingerprint composed of a multitude of vibrational bands, containing chemical information [7,66]. We used the quantum cascade laser (QCL) microspectrometer Spero-QT 340 and Chemical Vision software version 3.2 (Daylight Solutions, San Diego, USA). The instrument covers a spectral range from 1800 cm^−1^ to 948 cm^−1^ with a spectral resolution of 2 cm^−1^. Using the 4× objective (0.3NA), a Field of View of 2 × 2 mm is projected onto a 480 × 480 pixel microbolometer focal plane array (FPA) detector, resulting in an image with 4.25 × 4.25 μm pixel size. Before spectral measurements, tissue samples were thawed in a container purged with dry air. The dried samples were then placed in the cavity of the Spero-QT. The instrument was continuously purged with dry air during measurements.

**Immunohistochemical staining against Aβ (anti-Aβ IHC).** Following spectral measurements, sections were fixated in ethanol for 10 min and blocked for endogenous peroxidase using 0.3 % H_2_O_2_ for 5 min. After washing (3 × 3 minutes in PBS (Thermo Fisher)), the sample was incubated with the primary antibody mouse-anti-Aβ directed against aa1-16 (clone IC16, Müller-Schiffmann [67]) overnight. After washing (3 × 3 minutes in PBS), the sample was incubated with *Envision* (*Agilent Dako*) for 1 h at room temperature and washed. Color development was done using 3,3′ Diaminobenzidine (*Agilent Dako*), following the manufacturer's instructions. The sections were counter-stained with 1 % Cresyl-Violet in 50 % ethanol. Finally, the sections were dehydrated in an ethanol series (70%-96%–100 %), mounted with Euporal (Roth) and coverslipped. The stained sample was subsequently imaged with an *Olympus BX61VS* slide scanner, using the *UPlanSApo 20×0*.75 NA objective (Olympus).

**Image alignment and data selection.** In order to use anti-Aβ IHC images as ground truth of our dataset, they were precisely overlaid with QCL-IR images of the identical section. We applied a homemade software (written in *Matlab*) that warps the anti-Aβ IHC image onto the QCL-IR image, as previously described [7]. Subsequent to image registration, ROIs were manually selected, using a home made software, written in *Matlab*. ROIs are of size 272 × 272 μm, corresponding to 64 × 64 QCL-IR pixels. Aβ-positive ROIs contain at least 5 % of Aβ-positive area. Aβ-free ROIs were collected in the gray and white matter of AD cases, as well as healthy control cases. Additionally, ROIs were selected in tissue folds, dirt, edges of tissue and holes in the sample in order to represent a variety of different tissue structures.

**Data preprocessing.** For every ROI, a binary mask was calculated so that Aβ-positive pixels and their direct neighbors are represented as 1 and the surrounding areas as 0. In the absence of any Aβ deposits, a pixel value of 1 was assigned to the entire ROI to prevent divisions by zero and a vanishing gradient in the training process. The mask calculation was performed by using the blue color channel of the IHC images and adjusting the contrast such that the intensity values between 0.3 and 0.9 are mapped to a new lower and upper bound of 0 and 1. Values below or above the thresholds were clipped. Afterwards, the images were binarized using Otsu's method [68] and holes were filled. Connected components smaller than 542 μm^2^ (<30 QCL-IR pixel) were removed and binary objects were smoothed by a morphological opening. As a final step, ROIs were dilated with a 3 × 3 white kernel and 4 iterations, so that the pixels surrounding Aβ-structures are set to 1. All operations were performed using the image processing toolbox in Matlab (Matlab Image Processing Toolbox).

**Dataset:** The final dataset comprises 19 cases with a total of 12,630 ROIs, see Table 1. Training, validation and testing datasets were divided in a 10/5/4 split with an equal distribution of Aβ-positive and Aβ-free tissue tiles in each group. Training and validation data was used in the training process of the neural network while the testing data was utilized for independent evaluation. All measurements were strictly divided at case level such that data of the same case can only be found in one group.

### 4.1 Comparative Segmentation Network

To detect plaque related tissue structures, we trained a Comparative Segmentation Network (CompSegNet) [34] using the hyperspectral dataset described in Fig. 1. CompSegNet is a weakly supervised neural network that overcomes the need for pixel-precise annotation and only relies on image labels. The topology of the network is an extended U-Net [40] and follows the encoder-decoder structure (Fig. 3B). In the contracting path, an input image of size 64 × 64 × 427 is passed through a series of convolutional and max-pooling layers that reduces the spatial resolution while increasing the number of feature maps to a size of 8 × 8 × 512. Through a sequence of transposed convolutions, the spatial resolution is increased while the feature maps are reduced to match the number of output channels in the final layer. Encoder and decoder are connected through skip-connections to retain details of the input image. U-Net's output layer yields a bounded activation map within the interval [0,1] of the same spatial dimensions as the input image (64 × 64 × 1). The activation of each sample is then weighted by the corresponding binary mask, and averaged in a pooling neuron *q*, resulting in a ratio of associated Aβ activation within specific tissue areas. The percentage of activation is regulated by a transfer function, so that the activation for Aβ positive samples fall within a range of α and α+β and is minimized for control samples. An upper threshold is needed to prevent overdetection and ensure that only a fraction of the binary mask is activated. The overall loss is calculated by a cross entropy with class weighting on the output of the pooling neuron and an additional cross entropy that evaluates the activation pixel of the masked out Aβ-positive ROI and the ones that are fully Aβ-free (Further details in Schuhmacher et al., Section 2.3).

CompSegNet was trained on hyperspectral data for 500 epochs with an initial learning rate of 5 ·10^−4^, a decay of 0.9 every 50 epochs and early stopping as a regularizer, which stopped the training after 300 epochs. It was initialized with a lower bound of 5 % (α = .05) and an upper bound of 80 % (α + β = 0.8). We used a batch-size of 20 and RMSprop as the optimizer. To increase the size of the dataset, data augmentation strategies such as random rotation in steps of 90°, horizontal and vertical flipping were applied during training.

### 4.2 Model selection and evaluation

After training CompSegNet, a classification approach is utilized to evaluate each model on the validation dataset. Therefore, the averaged activation of the pooling neuron *q* is used to determine a predicted label for each ROI. If the relative amount of activation falls between the lower α and upper α + β bounds, the ROI is labeled as 1, and 0 otherwise. Metric values for each model were calculated and the model yielding the highest specificity values was chosen.

### 4.3 Whole-slide image segmentation

The QCL-IR image underwent a tile-based approach in which a sliding window of a fixed dimension of 64 × 64 pixel is moved across the image to extract tiles of the same size as the window. To ensure continuity of information across adjacent tiles, we created overlapping tiles of 16 pixels which are also assessed by the neural network. The resulting outputs are reassembled by selecting the maximum value of overlapping regions to form a WSI activation map.

### 4.4 Evaluation of segmentation performance

An ideal NN would generate an activation map that is identical to the corresponding binary IHC-mask of each QCL-IR ROI input. In other words, Aβ-positive pixels are 1 and Aβ-negative pixels are 0. In practice, the activation map spans values ranging from 0 to 1. In a well-trained NN, high values colocalize with Aβ-positive pixels, while low values colocalize with Aβ-negative pixels. We evaluate the NN's ability to segment plaques in QCL-IR images. Therefore, we quantify the segmentation accuracy in QCL-IR images from test cases. The generated activation maps are binarized and compared to the corresponding binary IHC-mask. The comparison is performed pixel by pixel, with each pixel classified as either *true positive (TP), false positive (FP), true negative (TN), or false negative (FN)*. Relevant metrics include the precision, also called *Positive Predictive Value (PPV)*

$$PPV=\frac{TP}{PP}=\frac{TP}{TP+FP}\text{,}$$

where Predicted Positives (PP) is the sum of all positively predicted pixels. The precision, in terms of application, expresses the proportion of truly Aβ-positive tissue in a plaque extraction. To put the precision into perspective, we consider the proportion of Aβ-positive area in a tissue section. This metric is known as *prevalence (PR)*, and it is defined as

$$\mathrm{PR}=\frac{P}{P+N}=\frac{TP+FN}{TP+FP+TN+FN}\text{,}$$

where *Positives (P)* and *Negatives (N)* denote the sum of actually positive/negative pixels. In terms of application, the prevalence expresses the proportion of Aβ-positive tissue in a random tissue extraction. The ratio of the two ratios explained above is a useful metric which we will refer to as *purification factor (PF)*

$$PF=\frac{PPV}{\mathrm{PR}}\;\text{.}$$

### 4.5 Plaque extraction via laser microdissection (LMD)

Laser microdissection (LMD) uses an ultraviolet laser to extract microscopic regions from a tissue section. Here it is used to extract plaques from brain tissue sections. The workflow is based on a previously described method [16,19]. QCL-IR images of brain tissue sections were measured using a Spero-QT as described above. The spectral data was subsequently processed by the CNN. Here, we use the activation map, the direct output of the CNN, as an indicator for plaques. “Plaque masks” were generated by binarizing the activation maps with a fixed threshold of 0.9 in a range of 0–1. Thereupon, the following morphological operations were applied in sequence: exclude objects with size <100 μmˆ2, dilate by 15 μm, fill holes, erode by 10 μm, exclude objects with size <300 μmˆ2, eccentricity <0.97, or solidity >0.7. The morphological processing and the coordinate transformation was performed in MATLAB. The resulting masks have reasonably plaque-like shapes and include a 5 μm margin to compensate for tissue loss during the LMD procedure. Damaged tissue regions were excluded manually. The sample was transferred to an LMD microscope (PALM MicroBeam; Zeiss, Jena, Germany). Based on three reference points in each microscopy image, a two-dimensional Helmert transformation was used to transfer coordinates of the detected plaque shapes. The PALM Robo software version 4.6 was used to import the plaque shape coordinates and cut the shapes using the instrument's 5× objective. The tissue was collected in dH_2_O and stored at 80 °C until analysis.

### 4.6 Sample preparation for proteomic analysis

Sample preparation was performed as previously reported [42]. In brief, samples were lysed in formic acid (FA) and sonicated. After evaporation (Concentrator plus; Eppendorf, Hamburg, Germany), an in-solution digestion with trypsin was performed after reduction and alkylation. The digestion was stopped by adding trifluoroacetic acid (TFA), and after another evaporation step, peptides were stored in 0.1 % TFA at −80 °C.

### 4.7 Mass spectrometric analysis

Liquid chromatography (LC) and mass spectrometry (MS) were carried out as described [69]. Briefly, an Ultimate 3000 RSLC nano LC system (Dionex, Idstein, Germany) coupled to an Orbitrap Fusion Lumos Tribrid mass spectrometer (Thermo Fisher Scientific) were used for the experiments. Peptides were separated at a flow rate of 400 nL/min with a linear gradient of increasing percentages of solution B (84 % acetonitrile, 0.1 % FA), starting at 5 % and increasing up to 30 % B at 105 min. The mass spectrometer operated in data-dependent acquisition mode, selecting all precursor ions with an intensity above 1 × 10^4^ for fragmentation at a fixed collision energy of 28 %. Precursor ions selected for fragmentation were maintained on a dynamic exclusion list for 30 s. The mass spectrometric data is available at the ProteomeXchange Consortium via the PRIDE partner repository [70] using the identifier PXD045130.

### 4.8 Analysis of proteomic data

Data obtained by MS were analyzed using MaxQuant [71] version: 2.0.3.0, with label free quantification (LFQ) and intensity-based absolute quantification activated. Trypsin was selected as the protease and a maximum of two missed cleavages were allowed. Obtained spectra were compared against a contaminant database and a homo sapiens reference proteome (January 2022) obtained from Uniprot [72]. The false discovery rate was set to 1 % and determined using a reverse-decoy database obtained from MaxQuant. Oxidation of methionine, carbamidomethylation of the N-terminus, deamidation of asparagine and glutamine and formylation of lysine were chosen as the dynamic modification, while carbamidomethylation of cysteine was used as the static modification. The obtained data from MaxQuant were subsequently analyzed with Perseus [73] by first filtering out decoys and contaminations and transforming the LFQ values (log2(x)). For further analysis, only proteins which could be identified in two of the three samples in each sample group were considered. Missing values were imputed using a normal distribution. A two-tailed Student's T-test was used to determine the significantly different proteins. Proteins with a p-value <0.05 were considered as significantly changed. Those proteins were divided into three groups (higher abundant post-IHC, not changed in abundance, higher abundant label-free) and solubility scores were calculated for each protein using Protein-Sol [43]. Solubility scores were tested for significant differences between two groups using a two-tailed Student's T-test. BoxPlot R [74] was used for the visualization of solubility scores.

## CRediT authorship contribution statement

**Dajana Müller:** Writing – review & editing, Writing – original draft, Visualization, Validation, Methodology, Formal analysis, Conceptualization. **Dominik Röhr:** Writing – review & editing, Writing – original draft, Visualization, Validation, Methodology, Formal analysis, Data curation, Conceptualization. **Baayla D.C. Boon:** Writing – review & editing, Data curation. **Maximilian Wulf:** Writing – original draft, Visualization, Methodology, Formal analysis. **Thomas Arto:** Writing – review & editing, Formal analysis, Data curation. **Jeroen J.M. Hoozemans:** Writing – review & editing, Data curation. **Katrin Marcus:** Writing – review & editing. **Annemieke J.M. Rozemuller:** Writing – review & editing, Data curation. **Frederik Großerueschkamp:** Writing – review & editing, Supervision. **Axel Mosig:** Writing – review & editing, Writing – original draft, Supervision, Methodology. **Klaus Gerwert:** Writing – review & editing, Writing – original draft, Supervision, Project administration.

## Ethics approval and consent to participate

Donors or their next of kin signed informed consent for the usage of brain tissue and clinical information for research purposes. The Institutional Review Board and Medical Ethical Board (METC) from the Vrije University Medical Center (VUmc) approved the procedures of the NBB.

## Consent for publication

All authors have read the manuscript and indicated consent for publication.

## Availability of supporting data

The data that support the findings of this study are available from the corresponding authors A.M. and K.G. upon reasonable request.

## Funding

This research was supported by the Protein Research Unit Ruhr within Europe (PURE), funded by the Ministry of Innovation, Science and Research (MIWF) of North-Rhine Westphalia, Germany (grant number: 233–1.08.03.03-031-68079), the Center for Protein Diagnostics (PRODI), funded by the Ministry of Culture and Science (MKW) of the State of North Rhine-Westphalia, Germany (grant number: 111.08.03.05–133,974), Alzheimer Nederland (WE.15-2019-13), and NIH (1R01AG061775).

## Declaration of competing interest

The authors declare the following financial interests which may be considered as potential competing interests: Prof. Dr. Klaus Gerwert reports financial support was provided by 10.13039/501100024593Ministry of Innovation, Science and Research (MIWF) and Ministry of Culture and Science (MKW) of the 10.13039/100030848State of North Rhine-Westphalia, Germany. Dr. Baayla D.C. Boon reports financial support was provided by 10.13039/501100010969Alzheimer Netherlands. The other authors declare that they have no known competing financial interests or personal relationships that could have appeared to influence the work reported in this paper.

## **Abbreviations**

AD: Alzheimer's disease
Aβ: amyloid beta
AUC-ROC: area under receiver operating characteristic curve
CompSegNet: Comparative Segmentation Network
CNN: convolutional neural network
FN: false negative
FP: false positive
FA: formic acid
IHC: immunohistochemistry
IR: infrared
LFQ: label free quantification
LMD: laser microdissection
LC: Liquid chromatography
MS: mass spectrometry
NIA-AA: National Institute on Aging-Alzheimer's Association
NBB: Netherlands Brain Bank
PET: polyethylene terephthalate
PPV: positive predictive value
PP: predicted positives
PR: prevalence
PF: purification factor
QCL: quantum cascade laser
ROIs: regions of interest
TFA: trifluoroacetic acid
TN: true negative
TP: true positive
