# Supplementary material for: Label-free Aβ plaque detection in Alzheimer's disease brain tissue using infrared microscopy and neural networks
Source: Heliyon. 2025 Jan 18;11(4):e42111. doi: 10.1016/j.heliyon.2025.e42111 (PMC11903818; doi:10.1016/j.heliyon.2025.e42111)
Supplement: Multimedia component 1 [file mmc1.docx]

**Supplementary Information of “Label-free Aβ plaque detection in Alzheimer’s disease brain tissue using infrared microscopy and neural networks”**

**Proof-of-concept with classic machine learning using a random forest (RF) approach**

In the preliminary phase of this study we aimed to answer two key questions: (i) whether QCL-IR spectra of plaques show distinct IR spectral patterns, and (ii) whether these patterns could effectively differentiate plaques from their surrounding tissue. For that purpose, a proof-of-concept study was conducted that aimed to distinguish QCL-IR spectra of plaques from spectra of surrounding tissue using machine learning.

Here, a brief explanation of the RF approach is provided. The reader is referred to previous publications by our group for a broader understanding of the application of supervised RF classifiers on IR images [1,2,3,4,5,6,7]. The RF database consists of labeled tissue-specific QCL-IR spectra. The RF is trained using this spectral database and ultimately produces classification maps that segment plaques from the surrounding tissue. A 100-tree RF ensemble with 16 nodes per tree and initially randomized absorbance-based binary conditions was constructed. Spectra are separated at each node based on whether they meet the binary criteria, allowing individual trees to classify each QCL-IR spectrum autonomously. The classification was finalized by a collective majority decision from all trees, delineating three distinct tissue types: (i) plaques, (ii) Aβ-free gray matter, and (iii) white matter. The QCL-IR spectra were first area-normalized, and then corrected for Mie scattering using RMieS-EMSC algorithm [8]. In the final step, the second derivative spectra were calculated using a Savitky-Golay filter with a 4th-order polynomial and 9 points window [9]. The training of the RF was performed on the preprocessed spectral data of 3 AD patients. To create the database for the RF, a binary mask for the Aß-positive pixels of the IHC was first created, analogous to the data preprocessing in the methods section. With this overlay, all plaque spectra could be automatically selected from the three AD patients. This was done analogously for surrounding tissues. Using the unsupervised K-means algorithm, analogous to the previous work, the plaque spectra and tissue spectra were classified into 50 similarity classes each and used as a training basis for the RF. To determine the accuracy of these classes and to correct outliers or mixed classes, false-color images were first generated on the three AD patients using the RF. The class assignment was checked by comparison with the IHC staining and corrected if necessary. Subsequently, the RF is validated on one control patient and five additional AD patients.

The classification maps derived from the RF are shown in Figure S1. Figure S1A1 shows the classification results on training data compared to the corresponding IHC image representations in Figure S1A2. Notably, the majority of plaques were segmented correctly from adjacent gray matter. Figure S1B depicts the RF's performance on test data. While several plaques were successfully identified, many others went undetected, and a few small regions were mistakenly identified as plaques or white matter. The RF shows limited detection of diffuse plaques, as shown in Figure S1C1. Figure S1C2 shows that even compact plaques exhibited partial identification. Notably, as shown in Figure S1C3, only the core, rather than the corona of classic cored plaques, was reliably detected. It is clear that the RF's performance varies significantly between the training and test datasets, indicating a significant issue with overfitting. However, expanding the training cohort in an attempt to address the aforementioned overfitting issue did not result in improvements in the RF's overall performance. This motivated us that a more complex model could fully exploit the potential of a larger cohort. In conclusion, training an image segmenting NN with an augmented dataset appeared to be a viable strategy for the advancement of label-free plaque detection within QCL-IR images.


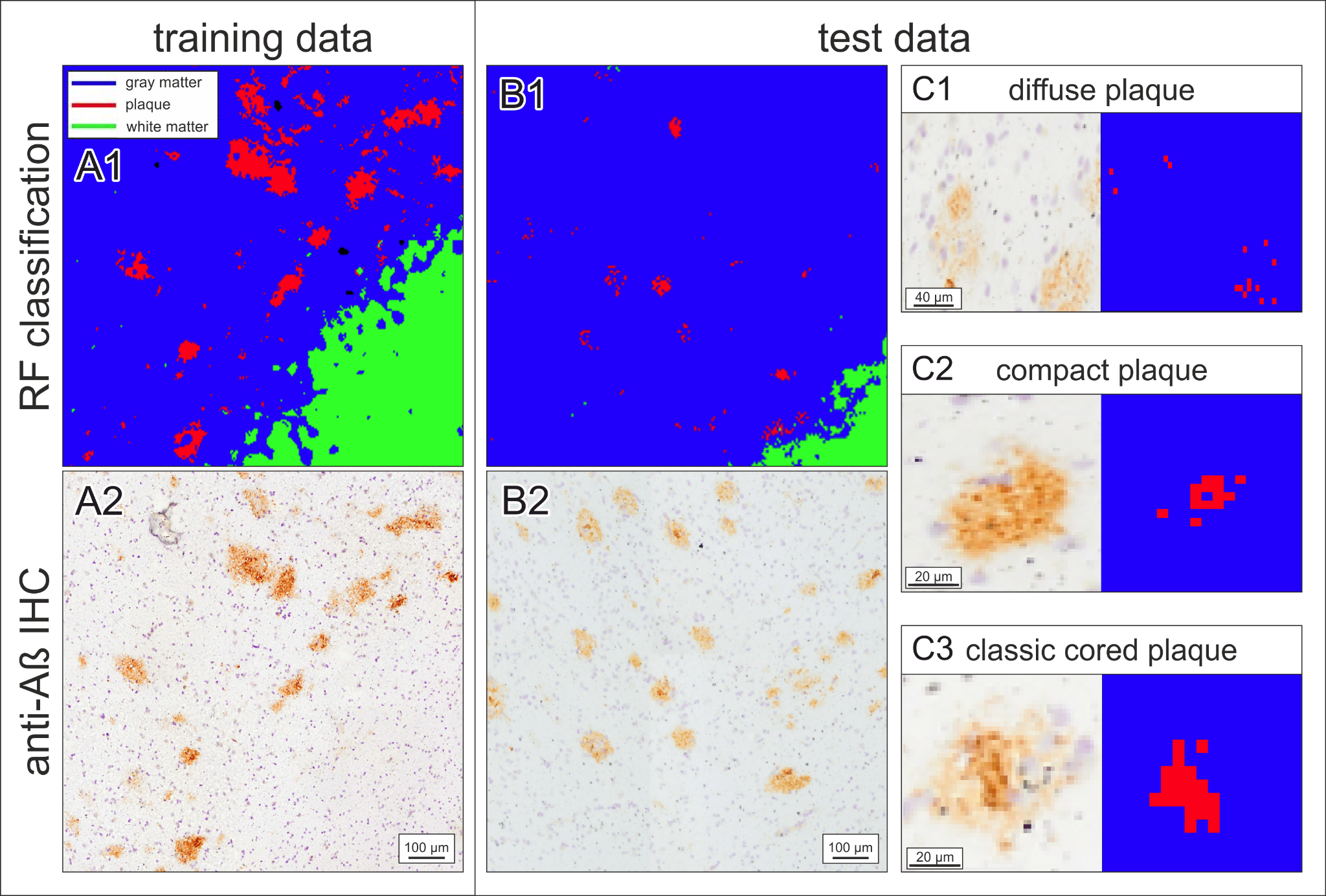


**Supplement Figure S1 Exemplary RF results** on **A** training data, and **B** test data. **C** Exemplary RF results on (1) diffuse, (2) compact, and (3) classic cored plaques.


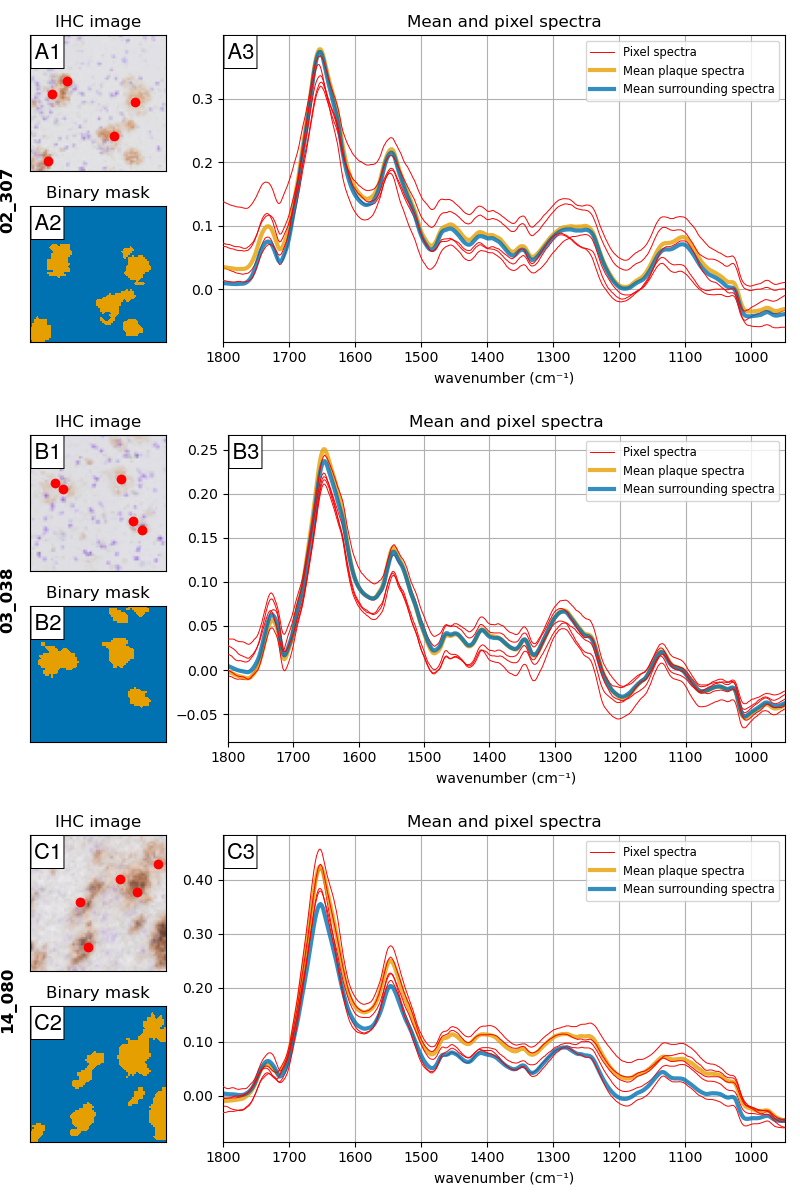


**Supplement Figure S2** Average and pixel spectra of ROIs per case. For each of the 3 cases, an Aβ-positive IHC image is shown (**A1**,**B1**,**C1**) with its corresponding binary mask (**A2**,**B2**,**C2**) (blue: Aβ-free; orange: Aβ-positive pixels). The average spectra of the binary masks are shown in **A3**, **B3** and **C3**, along with five individual pixel spectra in red. Red dots in the IHC image mark the positions of these pixels.


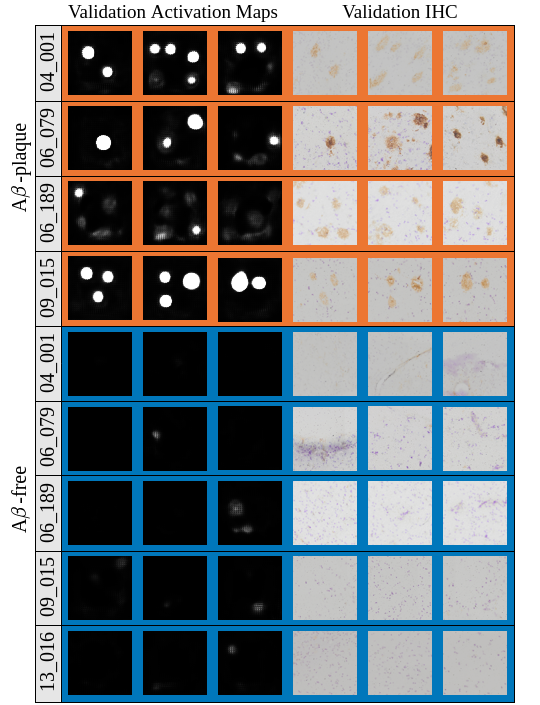


**Supplement Figure S3** Activation and IHC validation ROIs per case. Random selection of validation activation maps of Aβ-positive (orange) and Aβ-free (blue) and their corresponding IHC image for each case. Aβ-free ROIs were collected in the gray and white matter of AD cases, as well as healthy control cases.


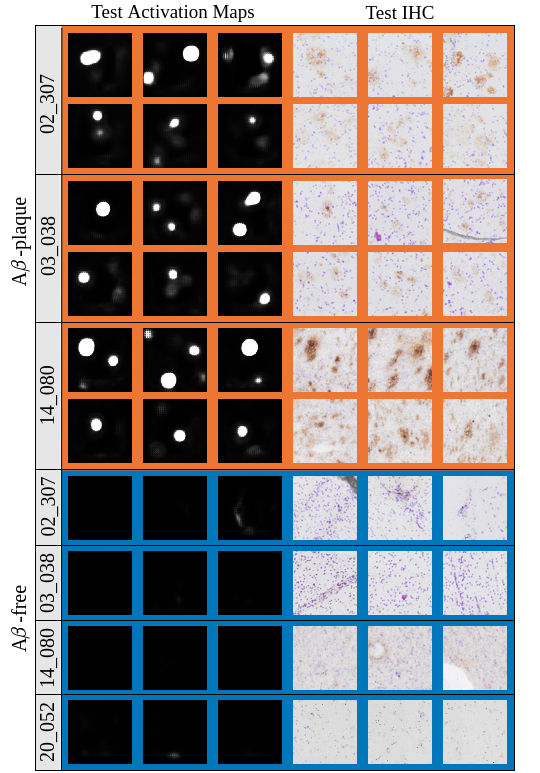


**Supplement Figure S4** Activation and IHC test ROIs per case. Random selection of test activation maps of Aβ-positive (orange) and Aβ-free (blue) and their corresponding IHC image for each case. Aβ-free ROIs were collected in the gray and white matter of AD cases, as well as healthy control cases.


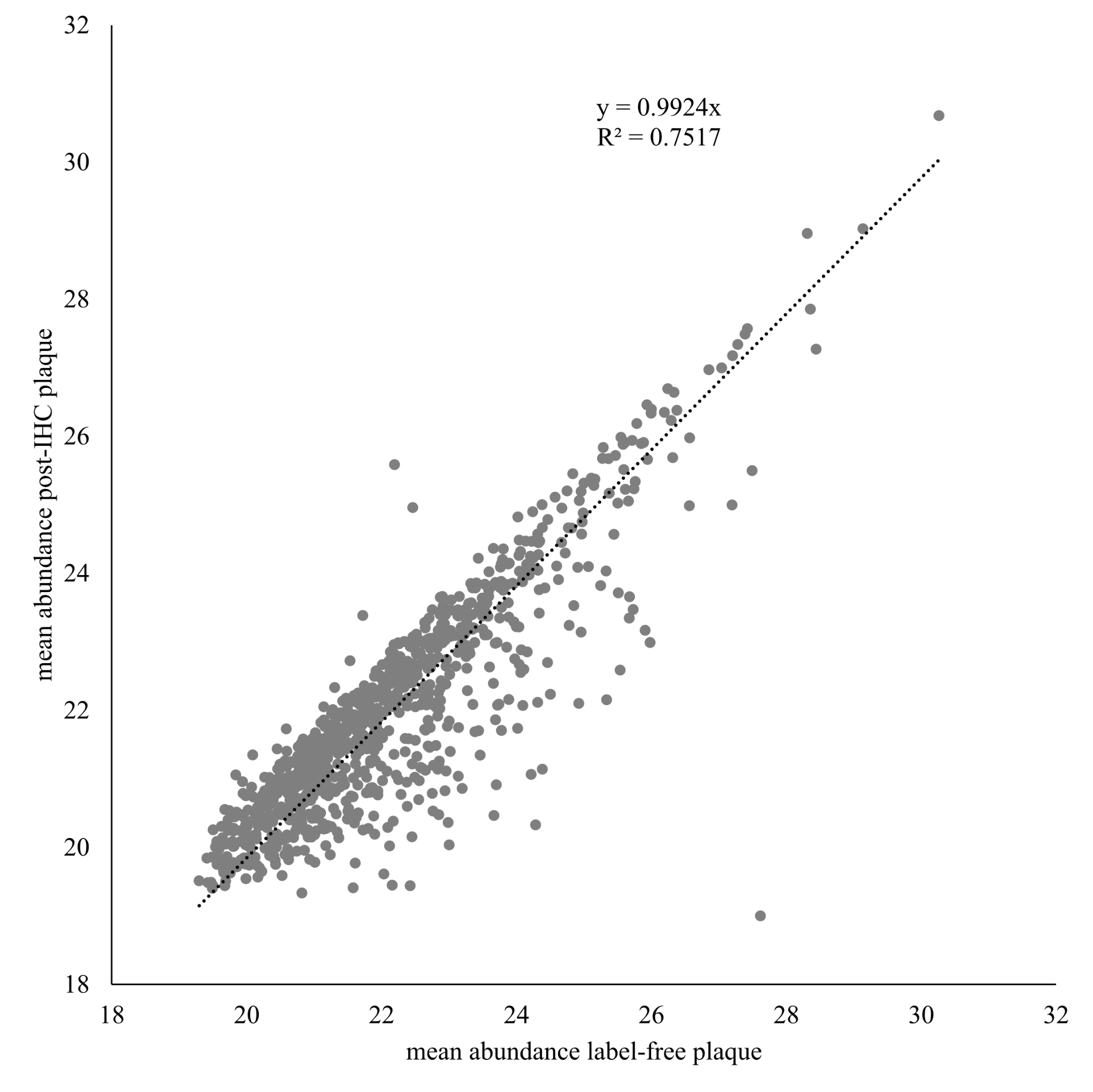


**Supplementary Figure S5** Mean LFQ values for proteins matching the criteria for the quantitative comparison (identified in two of three samples per group) were compared. The Pearson correlation equals 0.88, with a slope of 0.99 and an R^2^ value of 0.75, which indicates a good comparability of mean values for the majority of quantified proteins.


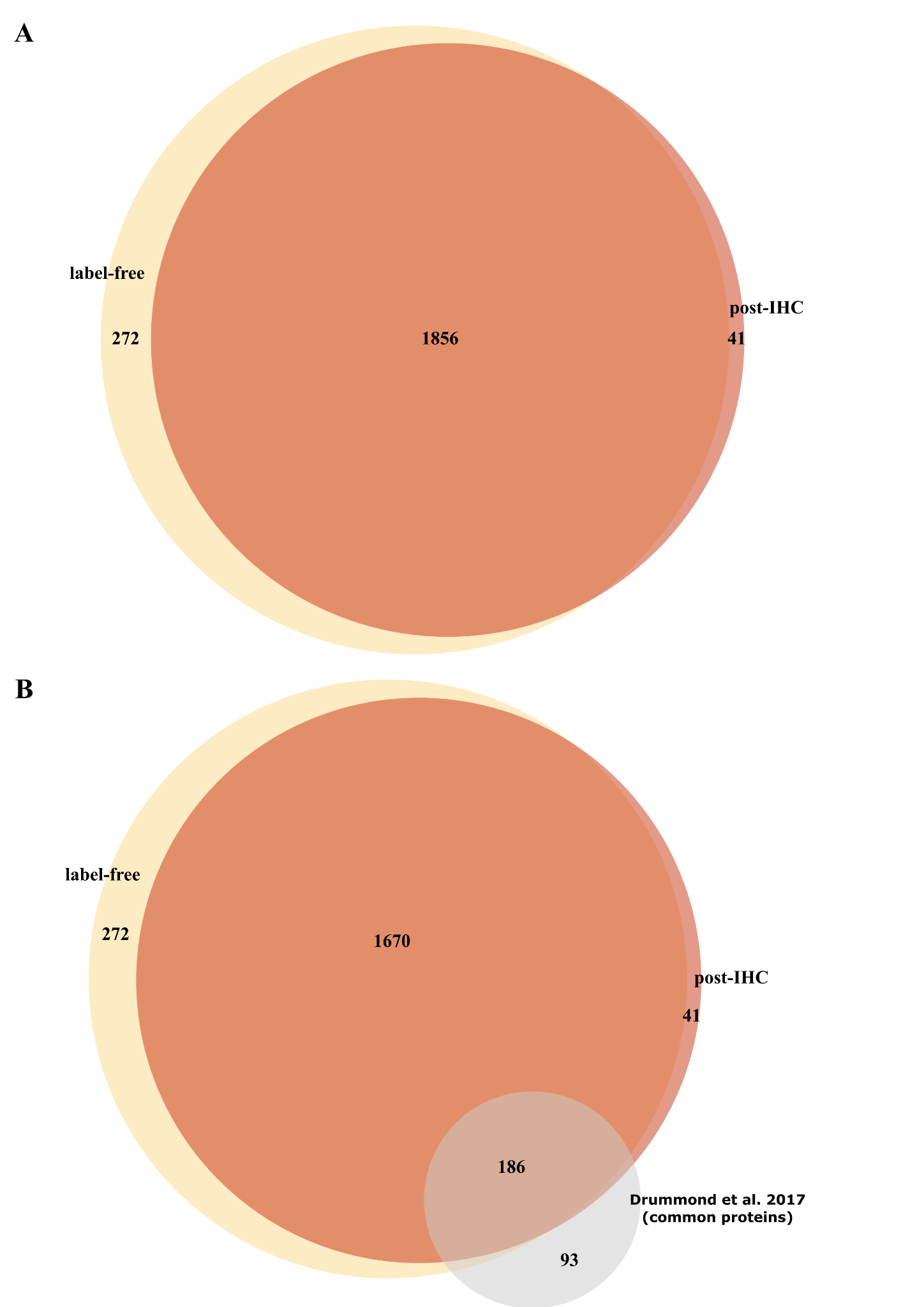


**Supplementary Figure S6** Venn diagrams showing the overlap in identified proteins between label-free (yellow) and post-IHC isolated plaques (red) in our study (A) and a comparison with a previously published data set by Drummond et al.. Venn diagrams were created by using DeepVenn [10].

**Supplementary Table ST1** Proteins exclusively identified in label-free plaques via bottom-up proteomics in sequential tissue sections of three AD cases within the independent test group.

| **Majority protein IDs** | **Protein names** | **Gene names** |
| --- | --- | --- |
| **Q96JH7** | **Deubiquitinating protein VCIP135** | **VCPIP1** |
| **D6RHE1;A0A5F9ZHE4;A0A5F9ZH30** | **Ankyrin 2** | **ANK2** |
| **Q96JM4;H0YEZ5** | **Leucine-rich repeat and IQ domain-containing protein 1** | **LRRIQ1** |
| **I3L0J9;Q6P2Q9** | **Pre-mRNA-processing-splicing factor 8** | **PRPF8** |
| **Q15120** | **[Pyruvate dehydrogenase (acetyl-transferring)] kinase isozyme 3, mitochondrial** | **PDK3** |
| **H0YCG0;A0A0G2JMS7;A0A0G2JNZ2;A0A0G2JPP5;A0A669KB89;Q14160** | **Protein scribble homolog** | **SCRIB** |
| **V9GY97;A0A087X0V7;A0A087WYH9;A0A075B7G8;J3KPL8;O00213** | **Amyloid beta A4 precursor protein-binding family B member 1** | **APBB1** |
| **Q15029** | **116 kDa U5 small nuclear ribonucleoprotein component** | **EFTUD2** |
| **Q9BT88** | **Synaptotagmin-11** | **SYT11** |
| **Q6NZI2** | **Polymerase I and transcript release factor** | **PTRF** |
| **A0A7I2V397;A0A7I2V5C6;A0A7I2V5H2;A0A7I2YQX5;A0A7I2YQT2;Q5JRX3;B4DEU0;B1APQ0;A0A7I2V5K2;A0A7I2V449;A0A7I2V4N3;A0A7I2V5R2;H0Y4F7;A0A7I2V3X0;A0A0A0MRX9;A0A7I2V3B6** | **Presequence protease, mitochondrial** | **PITRM1** |
| **A0A0A0MTC1;A0A0A0MTR7;Q63HN8** | **E3 ubiquitin-protein ligase RNF213** | **RNF213** |
| **F8VZY9;P05783** | **Keratin, type I cytoskeletal 18** | **KRT18** |
| **Q9BWH2** | **FUN14 domain-containing protein 2** | **FUNDC2** |
| **Q5TDF0;Q9BSD7** | **Cancer-related nucleoside-triphosphatase** | **NTPCR** |
| **Q9C0E8** | **Protein lunapark** | **LNP** |
| **A0A3B3IUC0;Q9Y287** | **Integral membrane protein 2B;BRI2, membrane form;BRI2 intracellular domain;BRI2C, soluble form;Bri23 peptide** | **ITM2B** |
| **K7EQA8;Q86X55** | **Histone-arginine methyltransferase CARM1** | **CARM1** |
| **P00451;B1B0G9;B1B0G8** | **Coagulation factor VIII;Factor VIIIa heavy chain, 200 kDa isoform;Factor VIIIa heavy chain, 92 kDa isoform;Factor VIII B chain;Factor VIIIa light chain** | **F8** |
| **Q5JW01;H0YCM7;Q5JVZ5;Q96JJ3** | **Engulfment and cell motility protein 2** | **ELMO2** |
| **H7C024;H7C410;P35052;C9J4Y6;H7BZE9;H7BZL4** | **Glypican-1;Secreted glypican-1** | **GPC1** |
| **P52788;H7C2R7** | **Spermine synthase** | **SMS** |
| **F8WAW4;F1LLU7;C9JB63;A0A0C4DGA2;O75521** | **Enoyl-CoA delta isomerase 2, mitochondrial** | **ECI2** |
| **E7EMB6;E5RIA4;E7ETB3;Q9ULA0** | **Aspartyl aminopeptidase** | **DNPEP** |
| **Q9Y6W5** | **Wiskott-Aldrich syndrome protein family member 2** | **WASF2** |
| **C9JD53;Q13907** | **Isopentenyl-diphosphate Delta-isomerase 1** | **IDI1** |
| **E9PMU7;E9PN18;A0A0J9YVR6;E9PQ56;A0A0J9YWM1;H0YEM1;A0A0J9YYL3;A0A0J9YXJ8;A0A0J9YVP6;A0A0J9YXX5;Q9UHX1** | **Poly(U)-binding-splicing factor PUF60** | **PUF60** |
| **K7EQ02;K7EQ55;K7EK33;Q96EP5** | **DAZ-associated protein 1** | **DAZAP1** |
| **C9JKG1;P21810** | **Biglycan** | **BGN** |
| **C9JKF7;C9JU59;E9PBD8;E7EMG9;P33241** | **Lymphocyte-specific protein 1** | **LSP1** |
| **Q99946** | **Proline-rich transmembrane protein 1** | **PRRT1** |
| **E9PQY2;Q9NQP4** | **Prefoldin subunit 4** | **PFDN4** |
| **P08294** | **Extracellular superoxide dismutase [Cu-Zn]** | **SOD3** |
| **Q5T4S7;X6R960;A0A0A0MSW0** | **E3 ubiquitin-protein ligase UBR4** | **UBR4** |
| **P04114** | **Apolipoprotein B-100;Apolipoprotein B-48** | **APOB** |
| **Q92556** | **Engulfment and cell motility protein 1** | **ELMO1** |
| **Q9H254;C9JY79;M0QZQ3** | **Spectrin beta chain, non-erythrocytic 4** | **SPTBN4** |
| **P11279** | **Lysosome-associated membrane glycoprotein 1** | **LAMP1** |
| **Q9NZN4** | **EH domain-containing protein 2** | **EHD2** |
| **P14543** | **Nidogen-1** | **NID1** |
| **A0A0G2JQM1** | **AP-2 complex subunit alpha-2** | **AP2A2** |
| **G5E9A7;Q09019** | **Dystrophia myotonica WD repeat-containing protein** | **DMWD** |
| **Q8TAF3** | **WD repeat-containing protein 48** | **WDR48** |
| **H0Y9G5;A0A669KAX3;O76024;A0A669KBF0** | **Wolframin** | **WFS1** |
| **Q66K74;M0QXQ9;M0R1J2;M0QY41** | **Microtubule-associated protein 1S;MAP1S heavy chain;MAP1S light chain** | **MAP1S** |
| **Q8TDQ7** | **Glucosamine-6-phosphate isomerase 2** | **GNPDA2** |
| **A0A6I8PLB1;A0A1B0GTF3;A0A6I8PIT5;A0A6I8PL32** | **Heat shock protein family A (Hsp70) member 12A** | **HSPA12A** |
| **A0A804CL36;Q13620;A0A7P0T9D0;K4DI93;A0A669KAX4;A0A7P0T9L3;A0A7P0T9C8;A0A7P0T8P8;A0A0A0MR50;A0A669KBG9;A0A7P0T9P5;A0A7P0TAF9;A0A7P0T9R8;A0A7P0T954;Q13619** | **Cullin-4B;Cullin-4A** | **CUL4B;CUL4A** |
| **P45954** | **Short/branched chain specific acyl-CoA dehydrogenase, mitochondrial** | **ACADSB** |
| **Q86UY8** | **5-nucleotidase domain-containing protein 3** | **NT5DC3** |
| **A0A384DVU0;Q8IY17** | **Neuropathy target esterase** | **PNPLA6** |
| **A6NKB8;Q9H4A4** | **Aminopeptidase B** | **RNPEP** |
| **P36222** | **Chitinase-3-like protein 1** | **CHI3L1** |
| **O95847** | **Mitochondrial uncoupling protein 4** | **SLC25A27** |
| **P22033** | **Methylmalonyl-CoA mutase, mitochondrial** | **MUT** |
| **P55884;C9JQN7;C9JZG1** | **Eukaryotic translation initiation factor 3 subunit B** | **EIF3B** |
| **Q9Y6X5;Q6UWV6** | **Bis(5-adenosyl)-triphosphatase ENPP4;Ectonucleotide pyrophosphatase/phosphodiesterase family member 7** | **ENPP4;ENPP7** |
| **Q96G03;E9PD70;E7ENQ8** | **Phosphoglucomutase-2** | **PGM2** |
| **E5RHJ4;G3V119;H0YB24;Q8N163** | **Cell cycle and apoptosis regulator protein 2** | **CCAR2** |
| **A0A494C143;Q14689;Q9P265** | **Disco-interacting protein 2 homolog A;Disco-interacting protein 2 homolog B** | **DIP2A;DIP2B** |
| **A0A087WXM8;P50895;K7ERB7;K7ENU8** | **Basal cell adhesion molecule** | **BCAM** |
| **E9PFZ2;P00450;H7C5R1** | **Ceruloplasmin** | **CP** |
| **Q68DH5** | **LMBR1 domain-containing protein 2** | **LMBRD2** |
| **O00401** | **Neural Wiskott-Aldrich syndrome protein** | **WASL** |
| **P05937** | **Calbindin** | **CALB1** |
| **J3KRL0;K7EIG7;J3QRN6;O94832** | **Unconventional myosin-Id** | **MYO1D** |
| **Q9Y426** | **C2 domain-containing protein 2** | **C2CD2** |
| **Q59FP8;Q92859** | **Neogenin** | **NEO1** |
| **Q9UN36;G3V2I9** | **Protein NDRG2** | **NDRG2** |
| **A0A6Q8PFC6;A0A6Q8PHC6;A0A6Q8PGA2;P50440** | **Glycine amidinotransferase, mitochondrial** | **GATM** |
| **Q7Z2K8** | **G protein-regulated inducer of neurite outgrowth 1** | **GPRIN1** |
| **O15055** | **Period circadian protein homolog 2** | **PER2** |
| **Q9HAT2** | **Sialate O-acetylesterase** | **SIAE** |
| **Q9UMX5** | **Neudesin** | **NENF** |
| **Q9NPH2** | **Inositol-3-phosphate synthase 1** | **ISYNA1** |
| **I3L3H2;P38919** | **Eukaryotic initiation factor 4A-III;Eukaryotic initiation factor 4A-III, N-terminally processed** | **EIF4A3** |
| **A0A1B0GV05;Q684P5** | **Rap1 GTPase-activating protein 2** | **RAP1GAP2** |
| **Q9NZ56** | **Formin-2** | **FMN2** |
| **Q53S08;Q9H0N0** | **Ras-related protein Rab-6C** | **RAB6C** |
| **Q16566** | **Calcium/calmodulin-dependent protein kinase type IV** | **CAMK4** |
| **D6RCA8;D6RFG5;D6RA82;P12429** | **Annexin;Annexin A3** | **ANXA3** |
| **D6RCQ8;D6RF77;D6RIS6;A0A494C163;A0A494C099;A0A087WT27;A0A494C1Q4;J3KN95;A0A494C1E2;A0A494C0G1;H0Y987;O95394** | **Phosphoacetylglucosamine mutase** | **PGM3** |
| **P62714;E5RHC1** | **Serine/threonine-protein phosphatase 2A catalytic subunit beta isoform;Serine/threonine-protein phosphatase** | **PPP2CB** |
| **A0A3B3IRX8;E7EV01;O15484** | **Calpain-5** | **CAPN5** |
| **A5YM72** | **Carnosine synthase 1** | **CARNS1** |
| **Q14118** | **Dystroglycan;Alpha-dystroglycan;Beta-dystroglycan** | **DAG1** |
| **Q5TDH0** | **Protein DDI1 homolog 2** | **DDI2** |
| **P31431** | **Syndecan-4** | **SDC4** |
| **F8VZG5;A0A5F9ZHP2;F8VY04;A0A5K1VW67;F8W1A4;P54819** | **Adenylate kinase 2, mitochondrial;Adenylate kinase 2, mitochondrial;Adenylate kinase 2, mitochondrial, N-terminally processed** | **AK2** |
| **P26639** | **Threonine--tRNA ligase, cytoplasmic** | **TARS** |
| **O14672** | **Disintegrin and metalloproteinase domain-containing protein 10** | **ADAM10** |
| **Q5VST6** | **Alpha/beta hydrolase domain-containing protein 17B** | **ABHD17B** |
| **E7EW77;E7EP65;A0A0C4DG21;A0A7D9NKC8;F8WAL6;Q9NYB9;E9PEZ7;H0Y6B5** | **Abl interactor 2** | **ABI2** |
| **P27695** | **DNA-(apurinic or apyrimidinic site) lyase;DNA-(apurinic or apyrimidinic site) lyase, mitochondrial** | **APEX1** |
| **Q9BY32** | **Inosine triphosphate pyrophosphatase** | **ITPA** |
| **Q92520** | **Protein FAM3C** | **FAM3C** |
| **K7EIK7;O95834;K7EII6;A0A0C4DGQ7;C9JRL6** | **Echinoderm microtubule-associated protein-like 2** | **EML2** |
| **G3V154;F2Z2T0;Q3YEC7** | **Rab-like protein 6** | **RABL6** |
| **Q9UKU7** | **Isobutyryl-CoA dehydrogenase, mitochondrial** | **ACAD8** |
| **A0A3B3ITC2;Q13884** | **Beta-1-syntrophin** | **SNTB1** |
| **O75475** | **PC4 and SFRS1-interacting protein** | **PSIP1** |
| **O43427;E9PSD3;E9PJW6** | **Acidic fibroblast growth factor intracellular-binding protein** | **FIBP** |
| **E5RFP0;Q8WVJ2** | **NudC domain-containing protein 2** | **NUDCD2** |
| **Q08380** | **Galectin-3-binding protein** | **LGALS3BP** |
| **J3QT28;O43684** | **Mitotic checkpoint protein BUB3** | **BUB3** |
| **H0YFP0** | **Calcium dependent secretion activator** | **CADPS** |
| **A0A087WW40;Q9Y371** | **Endophilin-B1** | **SH3GLB1** |
| **Q63HM9** | **PI-PLC X domain-containing protein 3** | **PLCXD3** |
| **H0YLH9;Q14005** | **Pro-interleukin-16;Interleukin-16** | **IL16** |
| **F5GZY1;Q9H993** | **Protein-glutamate O-methyltransferase** | **ARMT1** |
| **Q96HN2;H0Y8B3** | **Putative adenosylhomocysteinase 3;Adenosylhomocysteinase** | **AHCYL2** |
| **P30837** | **Aldehyde dehydrogenase X, mitochondrial** | **ALDH1B1** |
| **K7EQB1;Q9UNK0** | **Syntaxin-8** | **STX8** |
| **P20810;E7ES10;A0A0C4DGB5;A0A6Q8PGN6;B7Z574;E7EVY3;A0A6Q8PG72;E9PCH5;A0A6Q8PGG5;E9PDE4;A0A6Q8PFE5;A0A6Q8PFF6;H0Y9H6;H0Y7F0;E7EQA0;H0YD33;E7EQ12;A0A6Q8PH20;A0A6Q8PFL0;A0A6Q8PFR3** | **Calpastatin** | **CAST** |
| **S4R3U6;F5GZ08;B7Z4G8;P51693;K7EMN4** | **Amyloid-like protein 1;C30** | **APLP1** |
| **Q5T8U5;O15260** | **Surfeit locus protein 4** | **SURF4** |
| **P10253** | **Lysosomal alpha-glucosidase;76 kDa lysosomal alpha-glucosidase;70 kDa lysosomal alpha-glucosidase** | **GAA** |
| **Q9UPY6** | **Wiskott-Aldrich syndrome protein family member 3** | **WASF3** |
| **X6RAC9;P47813;O14602** | **Eukaryotic translation initiation factor 1A, X-chromosomal;Eukaryotic translation initiation factor 1A, Y-chromosomal** | **EIF1AX;EIF1AY** |
| **P00966;Q5T6L5;Q5T6L6** | **Argininosuccinate synthase** | **ASS1** |
| **H0YMF9;H0YN81;Q9GZS3** | **WD repeat-containing protein 61;WD repeat-containing protein 61, N-terminally processed** | **WDR61** |
| **Q92506** | **Estradiol 17-beta-dehydrogenase 8** | **HSD17B8** |
| **H0YEU5;H0YCT5;H0YF10;H0YDK2;Q09028;Q5JP02;E9PC52;Q16576** | **Histone-binding protein RBBP4;Histone-binding protein RBBP7** | **RBBP4;RBBP7** |
| **Q9BV79;H3BM30** | **Trans-2-enoyl-CoA reductase, mitochondrial** | **MECR** |
| **F8W0P7** | **ATP synthase F1 subunit beta** | **ATP5B** |
| **O43809;H3BV41;H3BND3** | **Cleavage and polyadenylation specificity factor subunit 5** | **NUDT21** |
| **A0A087WTZ5;E9PJ81;E9PRQ7;Q04323** | **UBX domain-containing protein 1** | **UBXN1** |
| **B8ZZ77;H7BZ14;Q9H2H8** | **Peptidyl-prolyl cis-trans isomerase;Peptidyl-prolyl cis-trans isomerase-like 3** | **PPIL3** |
| **F5GXE0;H0YGE9;F5GXU0;F5H883;F5GX30;P20645** | **Cation-dependent mannose-6-phosphate receptor** | **M6PR** |
| **F8WF86;Q8TBC4** | **NEDD8-activating enzyme E1 catalytic subunit** | **UBA3** |
| **J3KMY5;H0YIZ1;G3V3E8;E7EMS2;G3V3D1;P61916;G3V2V8** | **Epididymal secretory protein E1** | **NPC2** |
| **O96007** | **Molybdopterin synthase catalytic subunit** | **MOCS2** |
| **E7EV41;P32418** | **Sodium/calcium exchanger 1** | **SLC8A1** |
| **D6RIU4;D6RBV2;Q12907** | **Vesicular integral-membrane protein VIP36** | **LMAN2** |
| **P36575;A0A087WWQ5;D6RCT3** | **Arrestin-C** | **ARR3** |
| **P10645** | **Chromogranin-A;Vasostatin-1;Vasostatin-2;EA-92;ES-43;Pancreastatin;SS-18;WA-8;WE-14;LF-19;Catestatin;AL-11;GV-19;GR-44;ER-37;GE-25;Serpinin-RRG;Serpinin;p-Glu serpinin precursor** | **CHGA** |
| **Q5SX86** | **Rab GDP dissociation inhibitor** | **GDI2** |
| **A0A024R6I7;A0A0G2JRN3** | **Alpha-1-antitrypsin** | **SERPINA1** |
| **A8MT20;P41594** | **Metabotropic glutamate receptor 5** | **GRM5** |
| **C9JRU6;C9J0B2;C9J8R4;C9JVE2;Q96GG9** | **DCN1-like protein;DCN1-like protein 1** | **DCUN1D1** |
| **Q15424;Q14151** | **Scaffold attachment factor B1;Scaffold attachment factor B2** | **SAFB;SAFB2** |
| **A0A0U1RQW2;A0A0U1RQH7;H0Y4X3;G3XAC6;Q14498** | **RNA-binding protein 39** | **RBM39** |
| **H0YHR8;A0A6Q8PFH2;A0A6Q8PG36;Q5JUV4;A0A6Q8PHB5;A0A6Q8PHP0;P49902** | **Cytosolic purine 5-nucleotidase** | **NT5C2** |
| **A0A2R8Y6J3;P46777;Q5T7N0** | **60S ribosomal protein L5** | **RPL5** |
| **Q9NTX5** | **Ethylmalonyl-CoA decarboxylase** | **ECHDC1** |
| **P61758** | **Prefoldin subunit 3** | **VBP1** |
| **P05060** | **Secretogranin-1;PE-11;GAWK peptide;CCB peptide** | **CHGB** |
| **E5RJL1;Q99884** | **Transporter;Sodium-dependent proline transporter** | **SLC6A7** |
| **P13521** | **Secretogranin-2;Secretoneurin;Manserin** | **SCG2** |
| **G3V588;G3V5A3;Q13572** | **Inositol-tetrakisphosphate 1-kinase** | **ITPK1** |
| **H3BNC9;P08708;H0YN88;A0A075B716** | **40S ribosomal protein S17** | **RPS17** |
| **O95057** | **GTP-binding protein Di-Ras1** | **DIRAS1** |
| **H7C0Q6;Q9C037** | **E3 ubiquitin-protein ligase TRIM4** | **TRIM4** |
| **B1AH49;P25325** | **Sulfurtransferase;3-mercaptopyruvate sulfurtransferase** | **MPST** |
| **A0A7I2V5P2;A0A7I2V5X9;A0A7I2YQQ3;Q92820** | **Gamma-glutamyl hydrolase** | **GGH** |
| **Q5TI65;A0A087WVZ0;Q9Y5U8** | **Mitochondrial pyruvate carrier 1** | **BRP44L;MPC1** |
| **Q969E2** | **Secretory carrier-associated membrane protein 4** | **SCAMP4** |
| **I3L0A0;Q13404;G3V2F7;A0A0A0MSL3** | **Ubiquitin-conjugating enzyme E2 variant 1** | **TMEM189-UBE2V1;UBE2V1** |
| **P41091;Q2VIR3** | **Eukaryotic translation initiation factor 2 subunit 3;Putative eukaryotic translation initiation factor 2 subunit 3-like protein** | **EIF2S3;EIF2S3L** |
| **I3L1U7;I3L0X9;I3L3T0;A0A0A6YYL4;Q9Y3D7** | **Coronin;Mitochondrial import inner membrane translocase subunit TIM16** | **PAM16;CORO7-PAM16** |
| **Q8N8N7;G3V3Y1;G3V2R9** | **Prostaglandin reductase 2** | **PTGR2** |
| **I3L2R3;I3L2T8;I3L533;I3L2R9;A0A0J9YY14;J3QSD2;J3QRZ1;A6NIZ0;I3L3G9;A0A6Q8PGV6;A0A6Q8PEY3;I3L522;Q9NXR1;Q9GZM8** | **Nuclear distribution protein nudE homolog 1;Nuclear distribution protein nudE-like 1** | **NDE1;NDEL1** |
| **A6NDG6** | **Phosphoglycolate phosphatase** | **PGP** |
| **Q9NRX4** | **14 kDa phosphohistidine phosphatase** | **PHPT1** |
| **F8W031;F8VXJ7;Q9Y2B0;F8W1K5;F8VP03** | **Protein canopy homolog 2** | **CNPY2** |
| **Q5QPM2;Q5QPM1;Q5QPL9;Q9UKM9;Q5QPM0** | **RNA-binding protein Raly** | **RALY** |
| **J3QQM9;J3QRJ1;J3QL71;Q96FV2** | **Secernin-2** | **SCRN2** |
| **P49441;C9J173;B8ZZF6;E7ET59;E7EUX4;E7ENF2** | **Inositol polyphosphate 1-phosphatase** | **INPP1** |
| **Q06323;H0YKK6;H0YLU2** | **Proteasome activator complex subunit 1** | **PSME1** |
| **Q9P258** | **Protein RCC2** | **RCC2** |
| **P16930;A0A804HJ13;A0A804HJX2;H0YLC7;B7Z4W2** | **Fumarylacetoacetase** | **FAH** |
| **F8WE28;F8WE38;H7C0W6;Q9Y2C4** | **Nuclease EXOG, mitochondrial** | **EXOG** |
| **Q9BQI5;A0A804HJS7;A0A804HHZ7;A0A804HJJ5;A0A804HI01;A0A804HJG4;A0A804HHV6;A0A804HJI3;A0A804HJX5;A0A804HIU6;A0A804HJA8;A0A804HIX4;A0A804HHX3** | **SH3-containing GRB2-like protein 3-interacting protein 1** | **SGIP1** |
| **H0YNH0;H0YMB3;H0YLV5;A0A087WWM4;F8WAN9;H0YNJ6;Q9P2T1;A0A0B4J281;H0YMG3;H0YKK3;H0YN74;H0YNS9;H0YMR9;H0YMV5;H0YLB8;P36959** | **GMP reductase;GMP reductase 2;GMP reductase 1** | **GMPR2;GMPR** |
| **B4DE60;Q9BRK0** | **Receptor expression-enhancing protein;Receptor expression-enhancing protein 2** | **REEP2** |
| **O96008;K7EKG4** | **Mitochondrial import receptor subunit TOM40 homolog** | **TOMM40** |
| **P04181** | **Ornithine aminotransferase, mitochondrial;Ornithine aminotransferase, hepatic form;Ornithine aminotransferase, renal form** | **OAT** |
| **Q9UMX0;Q9NRR5** | **Ubiquilin-1;Ubiquilin-4** | **UBQLN1;UBQLN4** |
| **Q8N3F0** | **Maturin** | **MTURN** |
| **A0A6Q8PHF7;A0A1W2PR05;A0A6Q8PGS1;A0A1W2PS68;Q07699;B4DI92** | **Sodium channel subunit beta-1** | **SCN1B** |
| **H7BY36;A0A0D9SFL3;B0QYK0;C9JGE3;Q01844** | **RNA-binding protein EWS** | **EWSR1** |
| **P01303** | **Pro-neuropeptide Y;Neuropeptide Y;C-flanking peptide of NPY** | **NPY** |
| **Q9Y2Q5** | **Ragulator complex protein LAMTOR2** | **LAMTOR2** |
| **Q9NQR4;H7C579;F8WF70** | **Omega-amidase NIT2** | **NIT2** |
| **J3QLE5;P63162;P14678** | **Small nuclear ribonucleoprotein-associated protein N;Small nuclear ribonucleoprotein-associated proteins B and B** | **SNRPN;SNRPB** |
| **A0A024R161;P50151** | **Guanine nucleotide-binding protein subunit gamma;Guanine nucleotide-binding protein G(I)/G(S)/G(O) subunit gamma-10** | **hCG_1994888;GNG10** |
| **A0A7P0Z4L7;A0A7P0T9U1;A0A7P0Z469;A0A7P0T9G9;A0A7P0T8X2;A0A7P0TBM7;A0A7P0T900;A0A7P0T989;A0A7P0TA25;A0A7P0TB41;A0A7P0TBB5;A0A7P0Z4R9;A0A7P0TAD4;A0A7P0TAT6;A0A7P0T9U4;A0A7P0T926;A0A7P0T8I6;Q9UBR2** | **Cathepsin Z** | **CTSZ** |
| **O15212;A2AB88** | **Prefoldin subunit 6** | **PFDN6** |
| **Q5JTJ3** | **Cytochrome c oxidase assembly factor 6 homolog** | **COA6** |
| **A0A0C4DFZ2;P15289** | **Arylsulfatase A;Arylsulfatase A component B;Arylsulfatase A component C** | **ARSA** |
| **A0A0U1RQT8;Q8TCZ2** | **CD99 antigen-like protein 2** | **CD99L2** |
| **Q13825** | **Methylglutaconyl-CoA hydratase, mitochondrial** | **AUH** |
| **H0Y8X4;O43598** | **2-deoxynucleoside 5-phosphate N-hydrolase 1** | **DNPH1** |
| **A0A7I2V484;A0A7I2V5M3;P07711;Q5T8F0;A0A7I2YQA2** | **Cathepsin L1;Cathepsin L1 heavy chain;Cathepsin L1 light chain** | **CTSL** |
| **A0A0U1RRL7** | **MMP24OS** | **Protein MMP24OS** |
| **A0A0C4DH25;P01619** | **Ig kappa chain V-III region B6** | **IGKV3D-20** |
| **P51858** | **Hepatoma-derived growth factor** | **HDGF** |
| **A6NFX8;Q9UKK9** | **ADP-sugar pyrophosphatase** | **NUDT5** |
| **P16870** | **Carboxypeptidase E** | **CPE** |
| **Q9UKY7;D6RFH2;D6RDN0;D6R9V8;H0Y8K3** | **Protein CDV3 homolog** | **CDV3** |
| **Q9GZT8;E7EXA3;Q6X734;B8ZZI0** | **NIF3-like protein 1** | **NIF3L1** |
| **Q9ULC3** | **Ras-related protein Rab-23** | **RAB23** |
| **A0A804HJM4;A0A804HL30;A0A804HHT7;A0A804HKI4;A0A804HKC5;Q99614** | **Tetratricopeptide repeat protein 1** | **TTC1** |
| **P22676;H3BN14** | **Calretinin** | **CALB2** |
| **M0R0F0;P46782;M0QZN2;M0R0R2** | **40S ribosomal protein S5;40S ribosomal protein S5, N-terminally processed** | **RPS5** |
| **P61960** | **Ubiquitin-fold modifier 1** | **UFM1** |
| **P02790** | **Hemopexin** | **HPX** |
| **H0YN26;P39687;O43423** | **Acidic leucine-rich nuclear phosphoprotein 32 family member A;Acidic leucine-rich nuclear phosphoprotein 32 family member C** | **ANP32A;ANP32C** |
| **E9PRE3;H0Y424;A0A0A0MTE2;E9PMT2;F8WD26;J3KP06;Q8WWI1** | **LIM domain only protein 7** | **LMO7** |
| **A0A0B4J1Z1;C9JAB2;Q16629** | **Serine/arginine-rich splicing factor 7** | **SRSF7** |
| **A6NGJ0;P51808;F2Z328** | **Dynein light chain Tctex-type 3** | **DYNLT3** |
| **K7ENI8;P22748** | **Carbonic anhydrase 4** | **CA4** |
| **K7ERG4;P62316** | **Small nuclear ribonucleoprotein Sm D2** | **SNRPD2** |
| **R4GMU8;E9PLX3;A0A0C4DGV4;O43504** | **Ragulator complex protein LAMTOR5** | **LAMTOR5** |
| **E9PR30;P62861** | **40S ribosomal protein S30** | **FAU** |
| **A0A286YEY4;P01859** | **Ig gamma-2 chain C region** | **IGHG2** |
| **Q9UD71;J3KT77;J3KSJ8** | **Protein phosphatase 1 regulatory subunit 1B** | **PPP1R1B** |
| **J3QQZ9;Q9NVS9;J3QQV6;A0A286YFL3** | **Pyridoxine-5-phosphate oxidase** | **PNPO** |
| **P06307** | **Cholecystokinin;Cholecystokinin-58;Cholecystokinin-58 desnonopeptide;Cholecystokinin-39;Cholecystokinin-33;Cholecystokinin-25;Cholecystokinin-18;Cholecystokinin-12;Cholecystokinin-8;Cholecystokinin-7;Cholecystokinin-5** | **CCK** |
| **Q9Y3E1;M0R0J3** | **Hepatoma-derived growth factor-related protein 3** | **HDGFRP3;HDGFRP2** |
| **C9IYE8;E7ESZ6;C9J8S0;C9J6A7;C9J9T0;C9IZU8;Q96AT9** | **Ribulose-phosphate 3-epimerase** | **RPE** |
| **Q5T7C4;P09429;B2RPK0** | **High mobility group protein B1;Putative high mobility group protein B1-like 1** | **HMGB1;HMGB1P1** |
| **P62304** | **Small nuclear ribonucleoprotein E** | **SNRPE** |
| **F8WES2;F2Z2F3;J3QSB7;B4DUC8;Q13126** | **Purine nucleoside phosphorylase;S-methyl-5-thioadenosine phosphorylase** | **MTAP** |
| **E5RGX5;Q93045** | **Stathmin;Stathmin-2** | **STMN2** |
| **Q9Y5Z4;Q5THN1** | **Heme-binding protein 2** | **HEBP2** |
| **K7ESJ4;K7EQA1;O14737** | **Programmed cell death protein 5** | **PDCD5** |
| **P03886** | **NADH-ubiquinone oxidoreductase chain 1** | **MT-ND1** |
| **Q9BUT1;D6R9P2** | **3-hydroxybutyrate dehydrogenase type 2** | **BDH2;SLC9B2** |
| **H0YKJ4;H0YK48;A0A494BZZ2;A0A0S2Z4G6;P09493;Q6ZN40** | **Tropomyosin alpha-1 chain** | **TPM1** |
| **P28838;H0Y9Q1** | **Cytosol aminopeptidase** | **LAP3** |
| **A0A1W2PQV5;A0A804HKJ2;A0A1W2PNM1;A0A0A0MSE2;A0A0D9SFP2;E9PF18;Q16836;A0A1W2PQC2;A0A1W2PRT2;A0A804HJW7;A0A1W2PQ78;A0A804HLB5** | **Hydroxyacyl-coenzyme A dehydrogenase, mitochondrial** | **HADH** |
| **Q6P587** | **Acylpyruvase FAHD1, mitochondrial** | **FAHD1** |
| **H0Y9Y1;A0A7I2V5Y3;D6RDG3;A0A7I2YQL2;A0A7I2V3T6;P20290** | **Transcription factor BTF3** | **BTF3** |
| **A0A024RBT2;Q9NR28** | **Diablo homolog, mitochondrial** | **DIABLO** |
| **P02763** | **Alpha-1-acid glycoprotein 1** | **ORM1** |
| **H3BQ52;H3BMD8;P56211;H3BTD3;A6NMQ3;Q5T5H1;O43768** | **cAMP-regulated phosphoprotein 19;Alpha-endosulfine** | **ARPP19;ENSA** |
| **Q4G0N4;B7Z8V7;A0A0C4DGV3** | **NAD kinase 2, mitochondrial** | **NADK2** |
| **A0A087WT59;P02766** | **Transthyretin** | **TTR** |
| **Q5TD07;P16083;A2A2U4;Q5TD05** | **Ribosyldihydronicotinamide dehydrogenase [quinone]** | **NQO2** |
| **J3KTL2;Q07955;J3KSR8** | **Serine/arginine-rich splicing factor 1** | **SRSF1** |
| **C9JIZ0;O95777;F2Z2Y6;C9JNV3** | **U6 snRNA-associated Sm-like protein LSm8** | **LSM8** |
| **Q9H1E3** | **Nuclear ubiquitous casein and cyclin-dependent kinase substrate 1** | **NUCKS1** |
| **F8VVM2** | **Solute carrier family 25 member 3** | **SLC25A3** |
| **E5RI99;P62888;A0A0B4J213;E5RJH3;A0A0C4DH44** | **60S ribosomal protein L30** | **RPL30** |
| **Q9Y5L4** | **Mitochondrial import inner membrane translocase subunit Tim13** | **TIMM13** |
| **Q9Y5J7;G3V2F3;A0A1W2PRH9;A0A1W2PQS5;G3V502** | **Mitochondrial import inner membrane translocase subunit Tim9** | **TIMM9** |
| **F8WBW6;Q13442** | **28 kDa heat- and acid-stable phosphoprotein** | **PDAP1** |
| **Q8WVC2;P63220;Q9BYK1** | **40S ribosomal protein S21** | **RPS21** |
| **H0Y9T8;B1AHC3;B1AHC4** | **PRR5-ARHGAP8 readthrough** | **PRR5-ARHGAP8** |
| **E9PIH9;Q8NEC6;H0YDX3;F8WA39;Q13613;F8W764;F8W8S8;H7BZJ1;H7C406;E9PPP8** | **Myotubularin-related protein 1** | **MTMR1** |
| **Q16698;E5RFV2** | **2,4-dienoyl-CoA reductase, mitochondrial** | **DECR1** |
| **P56181** | **NADH dehydrogenase [ubiquinone] flavoprotein 3, mitochondrial** | **NDUFV3** |
| **E5RIW3;E5RJD8;E5RHG6;O75347** | **Tubulin-specific chaperone A** | **TBCA** |
| **Q7L266** | **Isoaspartyl peptidase/L-asparaginase;Isoaspartyl peptidase/L-asparaginase alpha chain;Isoaspartyl peptidase/L-asparaginase beta chain** | **ASRGL1** |
| **Q5T123;Q9H299** | **SH3 domain-binding glutamic acid-rich-like protein 3** | **SH3BGRL3** |
| **A0A1W2PQV2;H3BUH9;P23434** | **Glycine cleavage system H protein, mitochondrial** | **GCSH** |
| **A0A0C4DFV9;Q01105;P0DME0** | **Protein SET;Protein SETSIP** | **SET;SETSIP** |
| **P53999** | **Activated RNA polymerase II transcriptional coactivator p15** | **SUB1** |
| **D6R9C5;P10451** | **Osteopontin** | **SPP1** |
| **Q8TEA8;A0A2R8Y6X2** | **D-tyrosyl-tRNA(Tyr) deacylase 1** | **DTD1** |
| **P49006** | **MARCKS-related protein** | **MARCKSL1** |
| **E5RH81;E5RHP7;P00915;E5RIF9;E5RG43;E5RGU8;E5RFL2;H0YBE2;E5RFE7** | **Carbonic anhydrase 1** | **CA1** |
| **A0A6E1ZGS3;Q8IZY2** | **ATP-binding cassette sub-family A member 7** | **ABCA7** |
| **A0A0A0MTI5;B8ZWD1;P07108** | **Acyl-CoA-binding protein** | **DBI** |
| **B1AH72;P20472;B8ZZ19;H0Y3U0** | **Parvalbumin alpha** | **PVALB** |
| **P62857** | **40S ribosomal protein S28** | **RPS28** |
| **S4R3I5;O95167** | **NADH dehydrogenase [ubiquinone] 1 alpha subcomplex subunit 3** | **NDUFA3** |
| **Q15847** | **Adipogenesis regulatory factor** | **ADIRF** |
| **O60888;C9IZG4** | **Protein CutA** | **CUTA** |
| **A0A087X0H8;Q15714** | **TSC22 domain family protein 1** | **TSC22D1** |
| **P62328** | **Thymosin beta-4;Hematopoietic system regulatory peptide** | **TMSB4X** |

**Literature Supplement**

[1] Kallenbach-Thieltges, A. *et al.* (2013) ‘Immunohistochemistry, histopathology and infrared spectral histopathology of colon cancer tissue sections’, *Journal of Biophotonics*, 6(1), pp. 88–100. doi: 10.1002/jbio.201200132.

[2] Kallenbach-Thieltges, A., et al, Label-free, automated classification of microsatellite status in colorectal cancer by infrared imaging. Sci. Rep. 2020 https://www.nature.com/articles/s41598-020-67052-z

[3] Großerueschkamp, F. *et al.* (2015) ‘Marker-free automated histopathological annotation of lung tumour subtypes by FTIR imaging’, *Analyst*. The Royal Society of Chemistry, 140(7), pp. 2114–2120. doi: 10.1039/C4AN01978D.

[4] Großerueschkamp, F. *et al.* (2017) ‘Spatial and molecular resolution of diffuse malignant mesothelioma heterogeneity by integrating label-free FTIR imaging, laser capture microdissection and proteomics’, *Scientific Reports*, 7(1), p. 44829. doi: 10.1038/srep44829.

[5] Kuepper, C. *et al.* (2016) ‘Label-free classification of colon cancer grading using infrared spectral histopathology’, *Faraday Discussions*. Royal Society of Chemistry, 187, pp. 105–118. doi: 10.1039/C5FD00157A.

[6] Kuepper, C. *et al.* (2018) ‘Quantum Cascade Laser-Based Infrared Microscopy for Label-Free and Automated Cancer Classification in Tissue Sections’, *Scientific Reports 2018 8:1*. Nature Publishing Group, 8(1), pp. 1–10. doi: 10.1038/s41598-018-26098-w.

[7] Goertzen, N. *et al.* (2021) ‘Quantum Cascade Laser-Based Infrared Imaging as a Label-Free and Automated Approach to Determine Mutations in Lung Adenocarcinoma’, *The American Journal of Pathology*. Elsevier, 191(7), pp. 1269–1280. doi: 10.1016/J.AJPATH.2021.04.013.

[8] Bassan, Paul, et al. "Resonant Mie scattering (RMieS) correction of infrared spectra from highly scattering biological samples." Analyst 135.2 (2010): 268-277.

[9] Savitzky, A., & Golay, M. J. (1964). Smoothing and differentiation of data by simplified least squares procedures. Analytical chemistry, 36(8), 1627-1639.

[10] Hulsen, Tim. "DeepVenn--a web application for the creation of area-proportional Venn diagrams using the deep learning framework Tensorflow. js." *arXiv preprint arXiv:2210.04597* (2022).
